# Longitudinal paired liver biopsies and transcriptome profiling in alcohol-associated hepatitis reveal dynamic changes in cellular senescence

**DOI:** 10.1136/gutjnl-2024-334094

**Published:** 2025-03-23

**Authors:** Daniel Rodrigo-Torres, Alastair M Kilpatrick, Sofia Ferreira-Gonzalez, Rhona E Aird, Stephen Rahul Atkinson, Victoria L Gadd, Tak Yung Man, Luke D Tyson, Gopal Krishna R Dhondalay, Nikhil Vergis, Gavin E Arteel, Mark R Thursz, Laura Martinez-Gili, Stuart J Forbes

**Affiliations:** 1 Centre for Regenerative Medicine, Institute for Regeneration and Repair, The University of Edinburgh, Edinburgh, UK; 2 Division of Digestive Diseases, Department of Metabolism Digestion and Reproduction, Imperial College London, London, UK; 3 Centre for Inflammation Research, Institute for Regeneration and Repair, The University of Edinburgh, Edinburgh, UK; 4 Section of Bioinformatics, Division of Systems Medicine, Department of Metabolism, Digestion and Reproduction, Imperial College London, London, UK; 5 Division of Gastroenterology, Hepatology and Nutrition, Department of Medicine, University of Pittsburgh, Pittsburgh, Pennsylvania, USA; 6 Pittsburgh Liver Research Center, University of Pittsburgh, Pittsburgh, Pennsylvania, USA

**Keywords:** ETHANOL, INFLAMMATION, ALCOHOLIC LIVER DISEASE, LIVER REGENERATION

## Abstract

**Background and aims:**

Alcohol-associated hepatitis (AH) is an acute form of alcohol-related liver disease (ALD) with high mortality rate. AH is histologically characterised by cellular processes, including steatosis, inflammation and cell death. Apoptosis is the most studied form of cell death in AH; however, the role of cellular senescence, another response to cellular injury, in AH is unknown. Here, we explore the mechanisms of ALD pathophysiology and describe the role of senescence in AH.

**Methods:**

We performed RNA sequencing and bioinformatics analysis of 0- and 28-day transjugular liver biopsies (n=65) from patients with AH participating in the IL-1 Signal Inhibition In Alcoholic Hepatitis (ISAIAH) clinical trial. Additional bioinformatics reanalysis of existing AH transcriptomic datasets was conducted to confirm our findings. We also performed multiomic analysis of an in vitro model of AH with ethanol-treated hepatocytes overexpressing ethanol-metabolising enzymes.

**Results:**

Our longitudinal analysis revealed that senescence and inflammation were reduced at transcriptomic level following AH resolution; the expression of hepatocyte markers was increased. We identified two senescence-associated protein complexes, cytochrome c oxidase and the proteasome, which may act as senescence-induction mechanisms. We confirmed that senescence markers and pathways were increasingly expressed in hepatocytes as ALD progressed towards AH; this was partially reversed following AH resolution. Our in vitro model revealed that ethanol directly induces senescence and was dependent on ethanol metabolism.

**Conclusions:**

Our results suggest a possible pathogenic role for senescence in AH and indicate cellular senescence as a potential therapeutic target in early ALD to limit AH severity.

**WHAT IS ALREADY KNOWN ON THIS TOPIC**

- Alcohol-associated hepatitis (AH) is histologically characterised by inflammation, fibrosis, ductular reaction and apoptosis. While cellular senescence, a state of irreversible cell cycle arrest, has been implicated in different acute and chronic liver diseases, its role in alcohol-related liver disease, specifically in AH, is still unknown.

**WHAT THIS STUDY ADDS**

- Our unique transcriptomic dataset deriving from longitudinal paired biopsies of patients with AH showed that senescence and senescence-associated markers are dysregulated correlating with AH severity and senescence markers expression decreases following partial AH resolution. We demonstrate that the expression of cellular senescence markers increases in AH, compared with normal livers and other liver aetiologies. Our data are also relevant in identifying senescence-related mechanisms potentially involved in the resolution of liver injury. Finally, in vitro data demonstrate that hepatocyte senescence is a direct effect of ethanol insult.

**HOW THIS STUDY MIGHT AFFECT RESEARCH, PRACTICE OR POLICY**

- Our study highlights the potential involvement of senescent cells in AH pathophysiology and suggests targeting senescence as a future therapeutic intervention to limit the severity of AH episodes.

## Introduction

 Alcohol-related liver diseases (ALDs) encompass a spectrum of pathophysiological states, from early steatosis to cirrhosis and hepatocellular carcinoma.^1^ During disease progression, acute episodes clinically characterised by jaundice and liver failure known as alcohol-associated hepatitis (AH) can occur and are associated with a high short-term mortality rate (around 30%).^2^ Current AH treatments are mainly glucocorticoid based but are ineffective long term as they suppress the immune system, exacerbating infection risk.^3^ Liver transplant is curative but limited due to organ shortage; new AH treatments are, therefore, required. Recent clinical trials have targeted alternative mechanisms/pathways,^4^ including interleukin 1 (IL-1) pathway inhibition to ameliorate patients’ inflammatory response, either via anti-IL-1β antibodies (Canakinumab; IL-1 Signal Inhibition In Alcoholic Hepatitis (ISAIAH)^5^) or IL-1R antagonists (IL-1Ra) (Anakinra; (AlcHepNet)^6^).

AH is histologically characterised by steatosis, inflammatory infiltration, fibrosis, ductular reaction expansion and cell death (mainly necrosis and apoptosis).^7 8^ While cell death mechanisms have been described,^9^ the role of cellular senescence, another response to cell injury and stress, has not been studied in AH.^10^

Cellular senescence, a state of permanent cell cycle arrest in which apoptosis is prevented, has been described in chronic liver diseases.^11^ Senescence plays a role in both hepatocyte-injury (eg, metabolic dysfunction-associated steatohepatitis or hepatitis C) and biliary-injury conditions (eg, primary biliary cholangitis),^12 13^ where it has been linked with impaired repair and regeneration.^14^ In the context of chronic liver diseases, a combination of constant turnover to replace damaged cells and a damaging environment impairs and eventually exhausts the cells’ capacity to replicate and regenerate. Thus, cells activate the senescent and apoptotic machinery in response to persistent exposure to injury.^15^

Although some mechanisms have been described to be involved in disease progression,^1^ the precise mechanisms involved in an ineffective repair remain undefined. The exact role of senescence in AH and its implication on disease progression and ductular cell expansion is, therefore, still undetermined.

Here, we explored the mechanisms of ALD pathophysiology and defined the role of senescence in AH using a combination of multiomics data from human liver biopsies and in vitro models. By generating a unique transcriptomic dataset using paired AH biopsies, we showed that senescence is present in AH and reduced at transcriptomic level after ethanol-induced injury resolution.

We identified a senescence signature associated with ALD progression and confirmed that a senescence response is directly induced in hepatocytes following ethanol exposure in vitro. Our results suggest that senescence is involved in ALD progression and, therefore, a potential target for future AH therapies.

## Methods

### ISAIAH clinical trial

The ISAIAH clinical trial (clinicaltrials.gov identifier NCT03775109) was led by Imperial College London; the study design was approved by the UK Health Research Authority (18/LO/0745). The trial was performed in accordance with the Declaration of Helsinki; written informed consent was obtained from each patient or their legal representative.^5^ Transjugular liver biopsies were extracted from patients with AH participating in the ISAIAH clinical trial and flash frozen. Biopsies were taken at baseline (d0) and 28-days post-treatment with either placebo or canakinumab (d28).

### Patient and public involvement

Patients or the public were not involved in the design, or conduct, or reporting, or dissemination plans of this research.

### ISAIAH liver biopsy RNA extraction

Total RNA from flashfrozen transjugular liver biopsies (n=66) was extracted using QIAzol lysis reagent protocol (Qiagen). RNA samples were treated with Turbo DNA-free Kit (Thermo Fisher) to remove genomic DNA following manufacturer’s guidelines. RNA was assessed on the Agilent 2100 Electrophoresis Bioanalyser Instrument (Agilent Technologies, Inc., #G2939AA) and RNA 6000 Nano chips (#5067–1511) for quality and integrity of total RNA, and then quantified using the Qubit 2.0 Fluorometer (Thermo Fisher Scientific, Inc., #Q32866) and the Qubit RNA broad range assay kit (#Q10210). DNA contamination was quantified using the Qubit dsDNA HS assay kit (#Q32854).

### ISAIAH liver biopsy RNA sequencing

Where possible, 10 ng of each total RNA sample was fragmented to a size appropriate for Illumina sequencing; first-strand cDNA was generated using the SMARTer Stranded Total RNA-Seq Kit v2—Pico Input Mammalian kit (Clontech Laboratories, Inc., #634411). Samples were grouped for library prep based on the amount of fragmentation required, as recommended in the protocol: n=8 samples were subject to 4-min fragmentation time; n=15 samples were subject to 3 min; remaining samples required no fragmentation. Illumina-compatible adapters and indices were then added via five cycles of PCR. AMPure XP beads (Beckman Coulter, #A63881) were used to purify the cDNA library. Depletion of ribosomal cDNA was performed using ZapR v2 and R-probes v2 specific to mammalian ribosomal RNA and human mitochondrial rRNA. Uncleaved fragments were enriched by 13 cycles of PCR before a final library purification using AMPure XP beads. Libraries were quantified with the Qubit 2.0 Fluorometer and Qubit dsDNA HS assay and assessed for quality and size distribution of library fragments using the Agilent Bioanalyser and DNA HS Kit (#5067–4626). One sample failed library prep and was not taken forward for sequencing.

To try and balance the libraries prior to sequencing, libraries were pooled and run on an Illumina iSeq 100. Data from this run were used to attempt to rebalance the pool. Sequencing was performed on the Illumina NextSeq 2000 platform (Illumina, Inc., #20038897) using NextSeq 2000 P3 Kit (200 cycles v3 (#20040560)). The rebalanced pool of n=65 samples was run over three P3 flow cells at the Edinburgh Clinical Research Facility, Western General Hospital, Edinburgh, UK. PhiX Control v3 (Illumina, Inc., #FC-110–3001) was spiked into each run at a concentration of 1% to allow troubleshooting in the event of any issues. RNA-seq data were subject to standard quality control, alignment and read counting, before being imported into R for downstream analysis. RNA-seq data were subject to a common bioinformatics preprocessing pipeline, with minor variation as described in the Supplementary Information.

The RNA-seq data generated in this study have been deposited in National Center for Biotechnology Information (NCBI) Gene Expression Omnibus (GEO) (SuperSeries GSE270043).

### Immunohistochemistry

3 or 4-µm AH and 4-µm cirrhotic or normal liver paraffin sections were treated with either prewarmed citrate pH 6.0 or Tris-EDTA pH 9.0 for 10–15 min for antigen retrieval. Samples were incubated for 15 min at room temperature with BLOXALL (Vector Laboratories) for endogenous blocking of peroxidase and alkaline phosphatase, washed three times with phosphate-buffered saline (PBS), incubated with avidin/biotin blocking kit (Biolegend) for 15 min each at room temperature, washed again three times with PBS and incubated for 60 min at room temperature with protein block (Abcam) for blockage of non-specific antibody binding. Primary antibodies were applied and incubated overnight at 4 °C in a humidified chamber, as stated in online supplemental table 1. Samples were then sequentially incubated with species-specific biotinylated antibodies (online supplemental table 1) to detect primary antibodies, VECTASTAIN ABC reagent, R.T.U (Vector Laboratories) and ImmPACT DAB Substrate Kit, Peroxidase (HRP) (SK-4105) (Vector Laboratories); washes with PBS were applied between each step. Samples were finally counterstained with haematoxylin. Isotype controls were used at the same concentration as the corresponding primary antibody.

### Immunofluorescence

3 or 4-µm AH liver paraffin biopsies were treated with 10 mM sodium citrate pH 6.0 or 10 mM Tris base, 1 mM EDTA pH 9.0 for antigen retrieval. Sections were blocked with protein block (Abcam) and incubated using antibodies listed in online supplemental table 1. Primary antibodies were detected using fluorescent-conjugated secondary antibodies (Alexa-488, 555, Invitrogen). Sections were mounted with 4′,6-diamidino-2-phenylindole (DAPI) containing media (DAPI Fluoromount-G, SouthernBiotech) to stain cell nuclei. For p21/Ki67 double staining, Vector TrueVIEW Autofluorescence Quenching Kit (Vector Labs, #SP-8400) was used according to kit instructions.

Isotype controls were used in the same concentration as the primary antibody.

### VL-17A cells

VL-17A cells, which permanently overexpress mouse alcohol dehydrogenase (Adh1) and human cytochrome P450 2E1 (CYP2E1),^16^ were cultured in VL-17A media: Dulbecco’s modified Eagle medium, high glucose, GlutaMAX Supplement, pyruvate (Thermo Fisher Scientific) supplemented with 10% fetal calf serum (FCS; Life Technologies), 50-µg/mL Gentamicin (Gibco), 1% Penicillin/Streptomycin (Invitrogen), 400-µg/mL zeocin (Thermo Fisher Scientific) and 400-µg/mL geneticin (Gibco). When cells were cultured in the presence of ethanol, 25 mM of HEPES (Life Technologies) was added to the media (VL-17A complete media). For RNA-sequencing (RNA-seq) studies, cells were cultured in 25 cm^2^ flasks, in VL-17A complete media containing 0.5% FCS in the presence/absence of 100 mM ethanol (VWR chemicals, 437 433T) for 48 hours. Cells were collected at the end of the experiment in RLT buffer (Qiagen) for RNA extraction. For proteomic studies, VL-17A cells were grown in VL-17A complete media (10% FCS) in the presence/absence of ethanol at 100 mM. After 48 hours, cells were snap frozen for proteomics.

### RNA extraction, RT-qPCR and gene expression analysis

For in vitro studies, RNA was extracted from samples collected in RLT buffer using RNeasy mini kit (Qiagen), following manufacturer’s instructions. An additional step of DNase treatment was performed in those samples subsequently sequenced. RNA concentration and quality were assessed using a Nanodrop ND-1000 spectrophotometer (Thermo Fisher). 500 ng of RNA were reverse transcribed to cDNA using Quantitect Reverse Transcription Kit (Qiagen). Real-time qPCR was performed on a LightCycler 480 II (Roche) using Qiagen QuantiTect commercial primers (online supplemental table 2). Gene expression was normalised using Glyceraldehyde 3-phosphate dehydrogenase as housekeeping gene.

### Statistics

GraphPad Prism software (v.10.0.0) and R (v.4.2.3) were used for statistical analysis. For p21+ and p53+ hepatocytes quantification in paired biopsies, paired t-test method or Kruskal–Wallis test for non-parametric data method were used, as appropriate. p21+ and Ki67+ hepatocytes quantification in different liver aetiologies was tested by one-way ANOVA. Statistical significance in RT-qPCR data was tested using a two-tailed unpaired student’s t-test for parametric data or Mann–Whitney U-test for non-parametric data, as appropriate. Statistical significance was assumed at p<0.05. Data are given as mean±SD unless, otherwise, specified.

Further details regarding materials and methods are provided in the Supplementary Information.

## Results

### Senescence marker expression decreases in patients with severe AH (sAH) following partial AH resolution

To investigate the senescence response within AH and into AH resolution, we performed bulk RNA-seq analysis of longitudinal biopsies from a subset of patients with sAH (n=32) enrolled in the ISAIAH clinical trial.^5^ These data afford a unique insight into the dynamic transcriptomic changes underlying resolution following an sAH acute episode. Patients received either Canakinumab (anti-IL-1β antibody) or placebo; biopsies were taken at baseline (d0) and 28 days post-treatment (d28; n=27 patients with paired samples).

**Figure 1:**
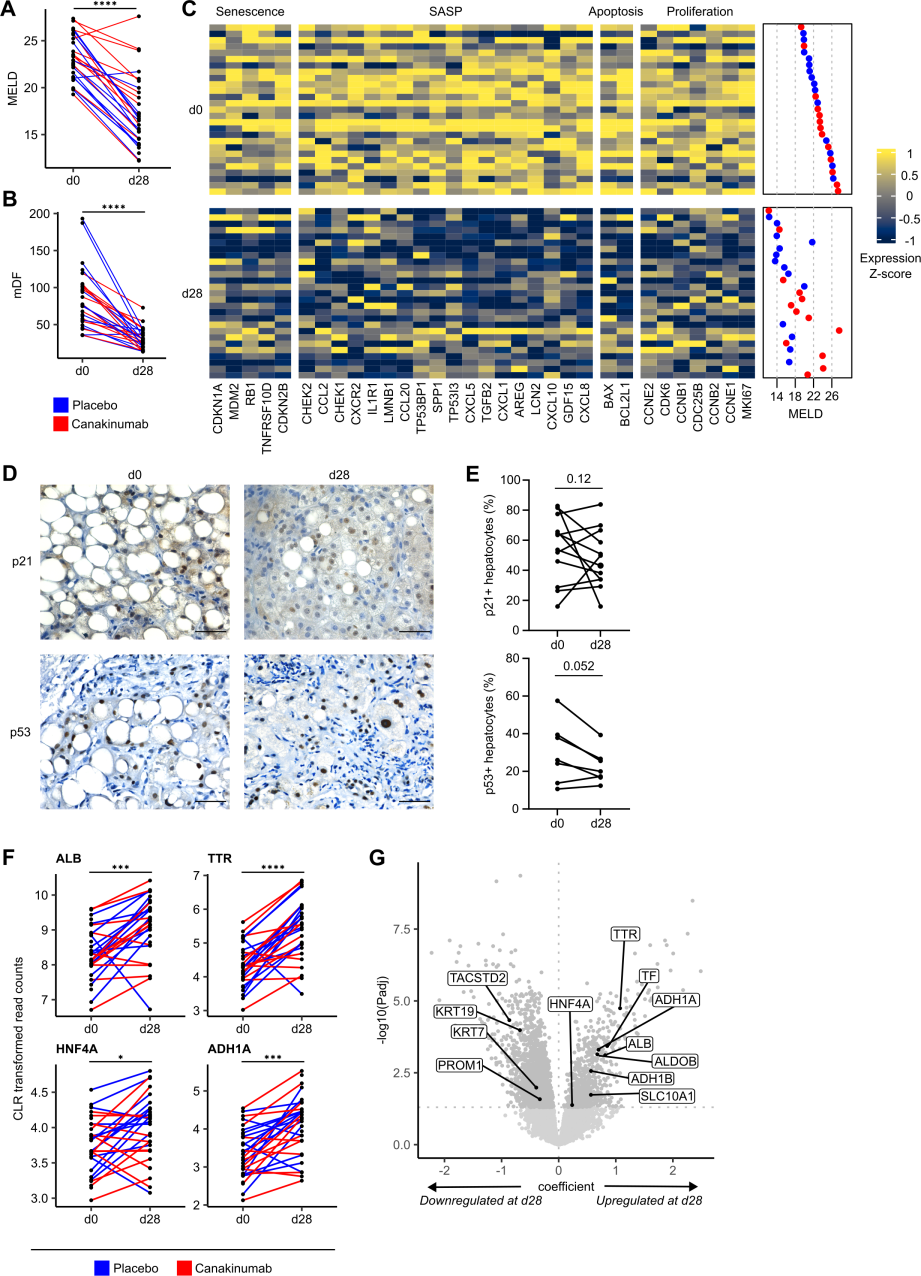
Senescence marker expression decreases in patients with sAH following AH resolution. Analysis of ISAIAH patients at d0 and d28. Points represent patients; lines connect matched measurements from the same patient, where available. (A) MELD measurements. (B) mDF measurements. (C) Heatmap of expression for significantly DE genes; at both timepoints, patients are ordered by increasing d0 MELD. (D) Representative staining of p21 and p53 in ISAIAH liver biopsies. (E) Quantifications of the percentage of p21+ and p53+ hepatocytes at d0 and d28 in paired biopsies (p21, n=12 paired biopsies and p53, n=7 paired biopsies). (F) CLR transformed read counts for hepatocyte markers. (G) Volcano plot of gene coefficient and significance between d0 and d28; points represent genes. Scale bar: 50 µm. *FDR<0.05; ***FDR<0.001; ****FDR<0.0001. AH, alcohol-associated hepatitis; DE, differentially expressed; FDR, false discovery rate; mDF, Maddrey’s discriminant function; MELD, model for end-stage liver disease; sAH, severe AH; SASP, senescence-associated secretory phenotype.

ISAIAH data indicated histological improvement of Canakinumab over placebo at d28, but no effect on clinical outcomes (eg, Lille score, Model for End-Stage Liver Disease score (MELD) and mortality).^5^ Almost all patients improved independent of treatment; of the full cohort (n=55), n=46 patients had decreased MELD at d28 (n=3 increased and n=6 incomplete data) and n=48 had decreased Maddrey’s discriminant function (mDF) (n=1 increased and n=6 incomplete data). The patient subset with RNA-seq data similarly improved (table 1): MELD and mDF fold change decreased; the placebo group showed slightly better improvement (figure 1A,B).

**Table 1:** ISAIAH patient data

	All patients with RNA-seq data				Patients with paired RNA-seq data (n=27)			
	Baseline (n=30)		d28 (n=29)		Baseline		d28	
	Placebo (n=16)	Canakinumab (n=14)	Placebo (n=16)	Canakinumab (n=13)	Placebo (n=14)	Canakinumab (n=13)	Placebo (n=14)	Canakinumab (n=13)
Age (mean, SD)	47.94, 7.11	50.64, 7.22	48.25, 7.95	50.77, 7.50	47.50, 7.37	50.77, 7.50	–	–
Sex (male/female)	7/9	9/5	6/10	9/4	5/9	9/4	–	–
90d mortality (died/alive)	1/15	0/14	1/15	0/13	1/13	0/13	–	–
MELD (mean, SD)	22.49, 2.17	23.65, 2.52	15.78, 2.53	19.17, 4.32	22.48, 2.33	23.87, 2.48	15.88, 2.58	19.17, 4.32
mDF (mean, SD)	89.32, 48.95	80.00, 25.25	27.06, 9.90	33.21, 17.23	90.98, 51.97	78.76, 25.84	27.94, 10.15	33.21, 17.23
Lille (mean, SD)	0.31, 0.24 (n=14)	0.28, 0.26 (n=13)	0.27, 0.24 (n=12)	0.26, 0.26 (n=12)	0.27, 0.24 (n=12)	0.26, 0.26 (n=12)	0.27, 0.24 (n=12)	0.26, 0.26 (n=12)
Lille responders (yes/no)	12/4	11/3	14/2	11/2	12/2	11/2	–	–
Fibrosis stage	Stage 3: n=15; no data: n=1	Stage 3: n=13; no data: n=1	Stage 3: n=16	Stage 3: n=10; no data: n=3	Stage 3: n=13; no data: n=1	Stage 3: n=12; no data: n=1	Stage 3: n=14	Stage 3: n=10; no data: n=3
Polymorphonuclear infiltration (yes/no)	8/8	7/7	4/12	1/12	6/8	7/6	2/12	1/12
Polymorphonuclear infiltration improvement (yes/no/no data)	7/8/1	6/8/0	5/8/3	6/7/0	5/8/1	6/7/0	5/8/1	6/7/0

mDF, Maddrey’s discriminant function; MELD, model for end-stage liver disease.

We found no significant transcriptomic differences associated with Canakinumab treatment. However, n=2921 genes were significantly differentially expressed (DE) among timepoints, independent of treatment, age or sex. We found significant downregulation of senescence markers, including cyclin-dependent kinase inhibitor (CDKN) 1A (CDKN1A; P21), CDKN2A (P16), CDKN2B (P15) and mouse double minute 2 homolog (MDM2) at d28 (figure 1C), independent of treatment (online supplemental figure S1A). Downregulation of the cell cycle arrest marker tumour protein P53 (TP53) was non-significant; however, the P53 pathway was significantly enriched (online supplemental figure S1B), with widespread downregulation of member genes (online supplemental figure S1C). Cyclins involved in G1 and G2 arrest were significantly downregulated at d28, as were markers of the senescence-associated secretory phenotype (SASP), a cocktail of secreted senescence factors, including chemokine (C-X-C motif) ligand 1 and amphiregulin (AREG) (figure 1C). SASP-related gene sets were significantly enriched, suggesting decreased expression of inflammatory cytokines and immune modulators following AH resolution. Signalling gene sets linked to senescence regulators, including Mammalian/mechanistic target of rapamycin complex (mTORC1) signalling^17^ and MYC proto-oncogene, bHLH transcription factor (MYC) targets^18^ were enriched. Together, these data suggest a pervasive reduction in senescence response following AH resolution.

Immunohistochemistry of ISAIAH biopsies showed a trend towards a decrease in the percentage of *p21*+ hepatocytes between d0 and d28 (figure 1D,E). *p53*+ hepatocytes followed a similar pattern, suggesting that despite significant transcriptomic differences, 28 days may not be sufficient to clear senescent cells from the liver.

AH is characterised by hepatic and systemic inflammation.^3^ Despite no significant differences associated with Canakinumab treatment, gene set enrichment analysis (GSEA) did show significant enrichment of gene sets and pathways relating to IL-1, specifically IL-1β (online supplemental figure S2A). While IL-1β was not significantly downregulated at d28, IL-1 receptor, type I and IL-1 receptor-like 2 were contributing to the enrichment of several IL-1β-related gene sets (online supplemental figure S2B). We found significant downregulation of plasmacytoid dendritic cell markers, including neuropilin 1 and S100 calcium-binding protein A8 (S100A8), at d28 (online supplemental figure S2C). GSEA showed significant enrichment of the acute inflammatory response gene set, driven by significant downregulation of several genes at d28, including S100A8 and inflammatory markers C reactive protein and CD163 (online supplemental figure S2D,E), indicating a reduction in inflammation following AH resolution.

Hepatocyte markers, including albumin (ALB), were significantly upregulated at d28 (figure 1F,G, online supplemental figure S2F); biliary epithelial cell (BEC) markers, including Keratins 7 and 19 and prominin-1, were significantly downregulated (figure 1G). To infer changes in cellular composition, we deconvolved our bulk RNA-seq data with a single-cell RNA-seq (scRNA-seq) reference dataset^19^ (online supplemental figure S3A). The predicted cellular composition showed a significant increase in the proportion of hepatocytes at d28, with decreases in proliferating cells and macrophages (online supplemental figure S3B); BECs were not present in this reference dataset. Further deconvolution with normal liver scRNA-seq data from the Liver Cell Atlas (LCA) also suggested an increase in the predicted proportion of hepatocytes and a decrease in BECs^20^ (online supplemental figure S3C,D). Gene module analysis with LCA data revealed that significantly upregulated genes were associated with hepatocytes; there was no strong cell type-specific signature among significantly downregulated genes (online supplemental figure S3E,F).

We observed enrichment of hypoxia (online supplemental figure S4A), a process previously implicated in ALD pathogenesis.^21^ There was significant downregulation of genes, including hypoxia-inducible factor 1-alpha at d28 (online supplemental figure S4B). Processes relating to cilium assembly and ciliopathies were also enriched (online supplemental figure S4C). Genes shared between these processes included several coding for dyneins and intraflagellar transport proteins (online supplemental figure S4D); we have previously shown that senescence and hypoxia are interlinked via the primary cilia in BEC in a liver transplant context.^22^

Caspases of the P53 signalling pathway leading to apoptosis were downregulated, although not significantly (online supplemental figure S1C). However, the apoptosis gene set was significantly enriched, with members significantly downregulated at d28. These included Tumor Necrosis Factor (TNF) receptor superfamily members 12A (TNFRSF12A; TWEAK receptor) and 10D (TNFRSF10D; Decoy receptor 2 (DCR2)), a pivotal factor between senescence and apoptosis.^23^

Downregulation of genes, including marker of proliferation Ki-67 (MKI67) and DNA topoisomerase IIα (TOP2A) at d28 (figure 1C, online supplemental figure S5A), suggests that, following peak injury, there is a reduction in the number of proliferating cells alongside the resolution of liver injury in AH. Similarly, we observed significant enrichment of fatty acid and bile acid metabolism gene sets (online supplemental figure S5B); members, including transthyretin (TTR), were increased at d28 (figure 1F). Together, these suggest liver metabolic function restoration following AH resolution.

Our data suggest that patient improvement following partial AH resolution is transcriptionally characterised by reduced expression of senescence, SASP and apoptosis-associated factors. We found no specific transcriptomic effect due to Canakinumab, but observed increases in hepatic and metabolic marker expression following partial AH resolution.

### Senescence-associated protein complexes are dysregulated with AH severity

**Figure 2:**
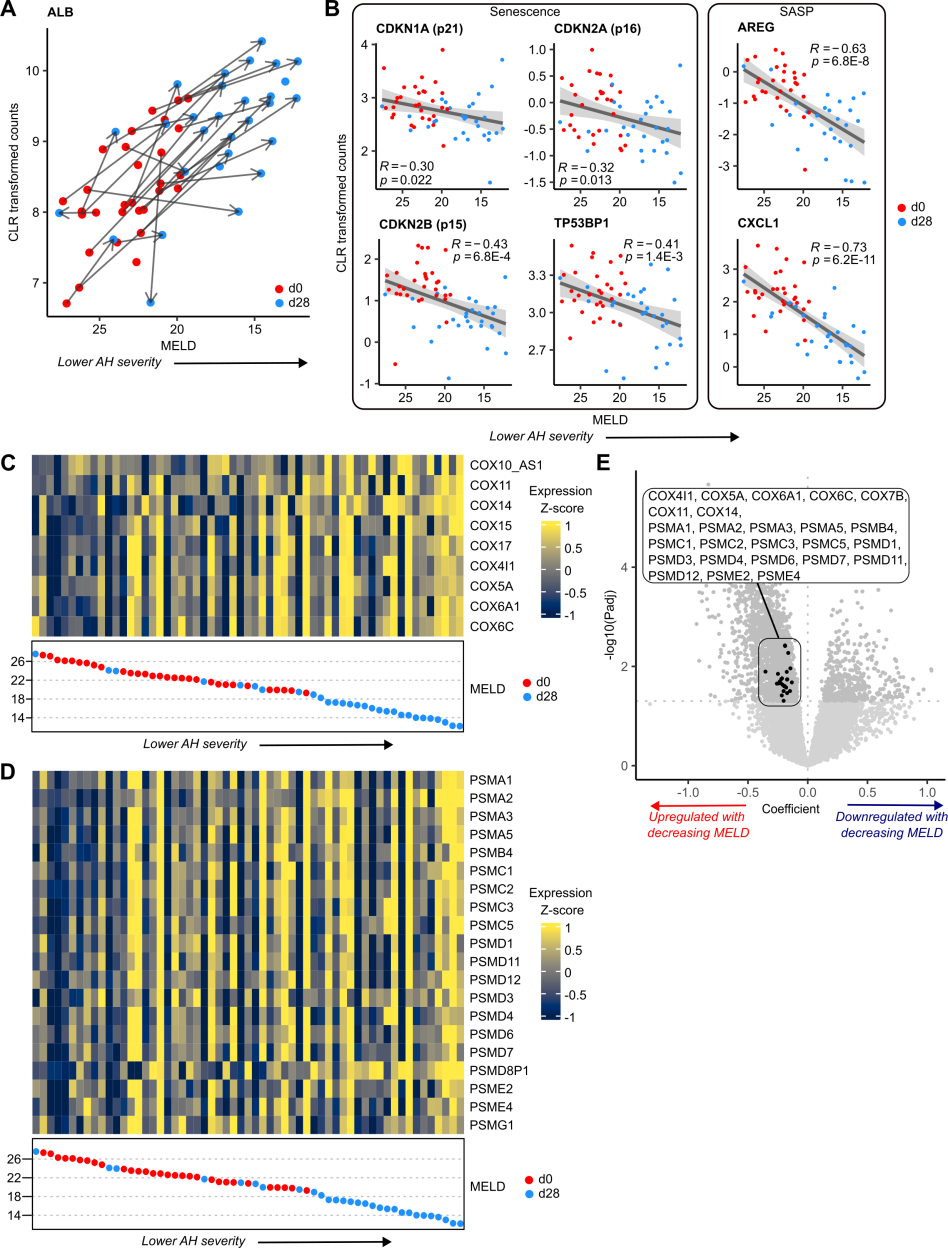
Senescence-associated protein complexes are dysregulated with AH severity. (A) CLR transformed read counts for ALB versus MELD, in ISAIAH patients, (d0, d28). Points represent patients; arrows connect matched measurements from the same patient, where available. (B) Correlation of CLR transformed read counts for senescence and SASP markers versus MELD, in ISAIAH patients, (d0, d28). Heatmaps of significantly DE (C) COX subunit and (D) proteasome subunit gene expression in ISAIAH patients. Patients are ordered by decreasing MELD; point colours represent timepoint. (E) Volcano plot of gene coefficient and significance with respect to MELD. Points represent genes; significantly DE COX and proteasomal subunit genes are highlighted. AH, Alcohol-associated hepatitis; ALB, albumin; COX, cytochrome c oxidase; DE, differentially expressed; MELD, model for end-stage liver disease; PSM, Proteasome; SASP, senescence-associated secretory phenotype.

To further investigate factors associated with AH severity in the ISAIAH cohort, we analysed transcriptomic changes with MELD; n=3557 genes were significantly DE. Since most patients’ MELD improved at d28, the results of this analysis naturally overlap with those from our time-based comparison. Hepatocyte markers, including ALB, were significantly upregulated as MELD decreased (figure 2A, online supplemental figure S6A). Genes upregulated with decreasing MELD were associated with hepatocytes (online supplemental figure S6B); downregulated genes were not cell-type specific (online supplemental figure S6C). The acute inflammatory response gene set was enriched (online supplemental figure S6D), driven by genes, including haptoglobin (online supplemental figure S6E), significantly upregulated with decreasing MELD. We found significant negative correlations between gene expression and MELD for senescence and SASP markers (figure 2B).

Interestingly, we also found genes important for core tissue functions associated with MELD, but not DE with time. Specifically, n=10 genes coding for cytochrome c oxidase (COX) complex (respiratory chain complex IV) subunits were upregulated with decreasing MELD (figure 2C), but not significantly DE with time. These COX subunits are involved in senescence-related metabolic pathways and gene sets, including two Reactome gene sets relating to TP53-mediated regulation (online supplemental figure S7A). Dysregulation of the COX complex may be mediated by mitochondrial dysfunction and significantly DE COX subunit genes are members of the significantly enriched mitochondrial complex IV assembly gene set (see Discussion). COX defects are linked to increased reactive oxygen species (ROS) production^24^; accordingly, our analysis showed a significant enrichment of ROS pathway in ISAIAH data (online supplemental figure S7B).

A second protein complex, the proteasome, is central to proteolysis and vital in maintaining cellular homeostasis. GSEA revealed significant enrichment of the proteasome (online supplemental figure S8A), driven by n=21 genes coding for proteasomal subunits significantly upregulated with decreasing MELD (figure 2D). None of these genes were significantly DE with time. Our data showed widespread upregulation of both core and regulatory proteasomal subunits (online supplemental figure S8B). These subunits are members of the MYC targets and mTORC1 signalling gene sets, as well as inflammatory response and IL-1 signalling gene sets. They also contribute to enrichment of gene sets associated with senescence markers and mechanisms, such as SCF(Skp2)-mediated degradation of p27/p21 and stabilisation of p53 (online supplemental figure S8C,D).

Cellular response to chemical stress was also significantly enriched. Significantly DE genes driving this enrichment included those coding for COX (n=7) and proteasomal (n=18) subunits (figure 2E), further suggesting that both complexes are dysregulated due to an ethanol-induced stress response. ALB and Ubiquitin B, a gene regulator which labels proteins for degradation by the proteasome, were also significantly DE members of this gene set.

Together, these data indicate a transcriptomic signature associated with MELD independent of time, which suggests roles for the senescence-related proteasome and COX protein complexes in AH; both complexes may be dysregulated as an ethanol-induced stress response.

### ALD progression is characterised by transcriptomic dysregulation of the proliferation/senescence axis

**Figure 3:**
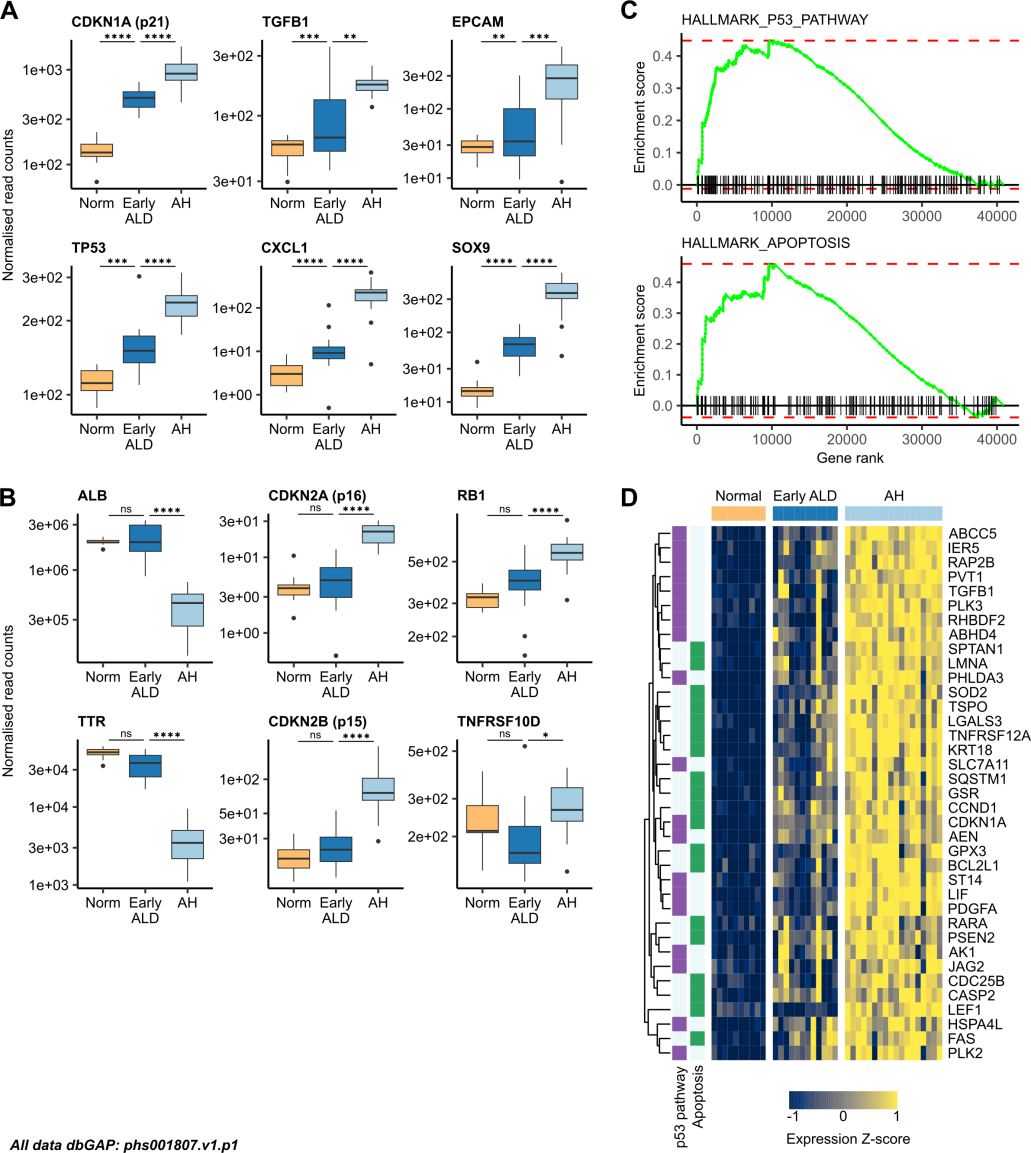
ALD progression is characterised by transcriptomic dysregulation of the proliferation/senescence axis. Boxplots of normalised read counts for (A) senescence, SASP and biliary markers; (B) hepatic and senescence markers in normal liver (n=10), early ALD (n=12) and AH (n=18). (C) GSEA for the MSigDB Hallmark P53 pathway and apoptosis gene sets, comparing AH to normal liver. (D) Heatmap of genes associated with the P53 pathway and apoptosis in normal liver, early ALD and AH. AH, alcohol-associated hepatitis; ALD, alcohol-related liver disease; GSEA, gene set enrichment analysis; SASP, senescence-associated secretory phenotype.

To get a wider picture of senescence gene dysregulation in ALD and AH, we reanalysed RNA-seq data from the InTEAM consortium.^25^ n=872 genes progressively increased their expression with ALD progression; they were significantly upregulated in both early ALD versus normal liver and AH versus early ALD (online supplemental figure S9A). This group contained markers of senescence (eg, CDKN1A and TP53) and SASP. We also found BEC markers (eg, SRY-Box transcription factor 9 and epithelial cell adhesion molecule) (figure 3A, online supplemental figure S9A). Overenrichment analysis identified processes including DNA damage response, tissue regeneration and regulation of apoptotic signalling pathway, driven by genes including CDKN1A, TP53, MDM2 and Bcl-2-like protein 1 (BCL2L1) (online supplemental figure S9B,C).

Hepatocyte markers ALB, TTR and alcohol dehydrogenase 1A (ADH1A) were not significantly DE between normal and early ALD but were significantly downregulated in AH versus early ALD, suggesting a secondary transcriptomic response following onset of liver failure (figure 3B). Other senescence markers (eg, CDKN2A, CDKN2B and TNFRSF10D) were upregulated in AH versus early ALD but not in normal versus early ALD (figure 3B). Many senescence-related genes were also upregulated in AH versus control groups whose liver disease was not alcohol related (eg, Metabolic dysfunction–associated steatotic liver disease (MASLD) and compensated HCV cirrhosis), suggesting an effect specific to alcohol-related injury (online supplemental figure S9D).

Markers of DNA damage (eg, tumour suppressor p53-binding protein 1 and checkpoint kinase 2), SASP (eg, AREG and IL-6) and inflammation (eg, secreted phosphoprotein 1 and Lipocalin-2 (LCN2)) followed the same expression pattern (online supplemental figure S9E).

Transition from normal liver to early ALD was characterised by significant upregulation of proliferation markers (eg, MKI67 and TOP2A); many of these were not significantly DE between AH and early ALD (online supplemental figure S9F).

GSEA of normal versus AH tissue revealed significant enrichment of the P53 pathway and apoptosis gene sets (figure 3C); n=37 genes were core enrichment drivers within the n=872 progressively upregulated genes identified above (figure 3D). A further n=62 genes were significantly upregulated in AH versus normal liver (online supplemental figure S10A). GSEA also revealed significant downregulation of metabolic factors in AH compared with normal liver, with enrichment of fatty acid and bile acid metabolism gene sets (online supplemental figure S10B).

**Figure 4:**
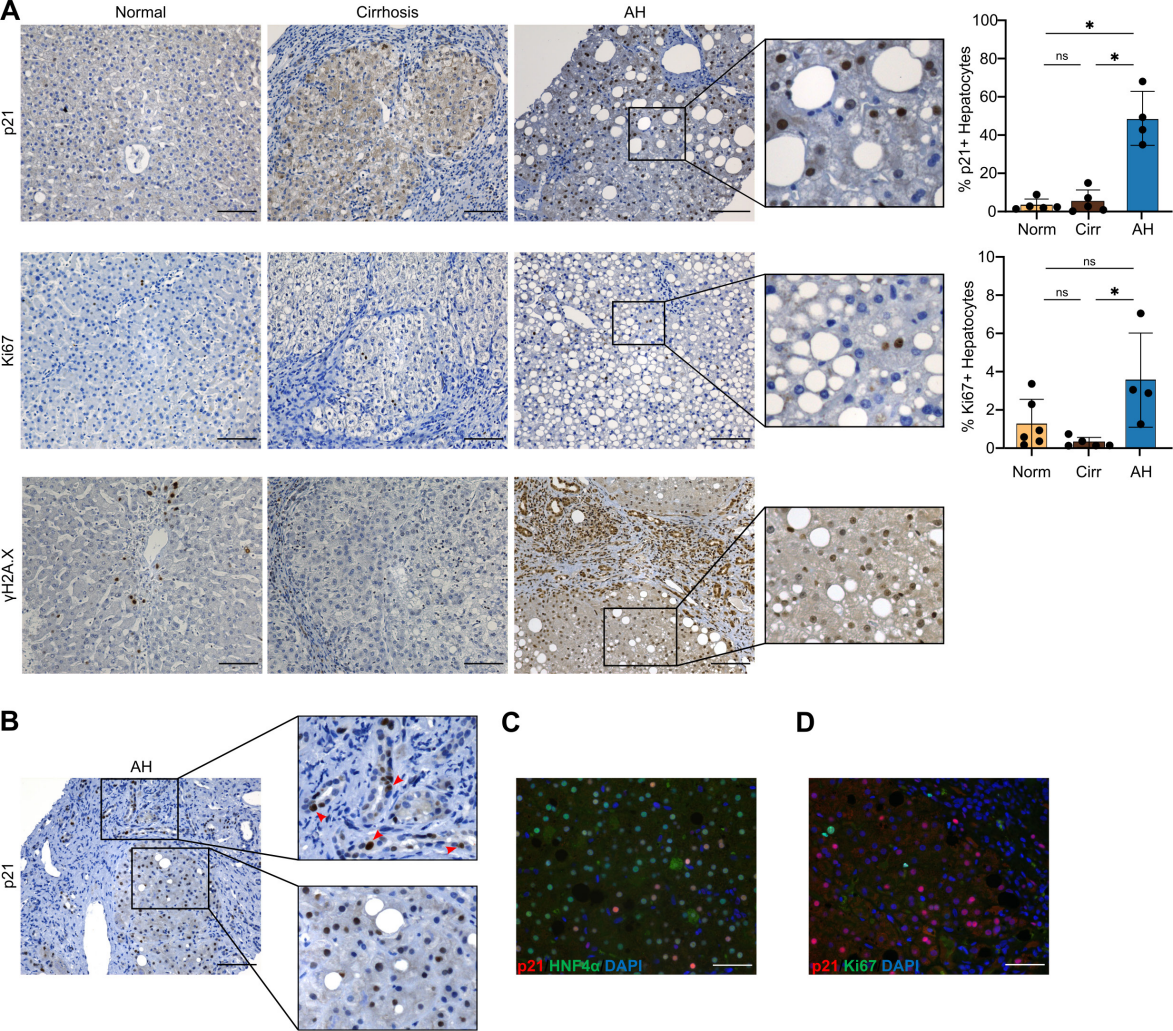
AH is characterised histologically by increased presence of p21-positive cells. (A) Representative staining of p21, Ki67 and γH2A.X in normal, cirrhotic and AH livers, including digital magnification of p21, Ki67 and γH2A.X stainings in AH. Far right, quantification of percentage of p21+ and Ki67+ hepatocytes in normal (n=5–6), cirrhotic (n=5) and (n=4) patients with AH (mean±SD). (B) Representative staining of p21 in AH liver biopsies. Top far right, digital magnification of area showing p21+BECs (red arrows), bottom far right, digital magnification of area showing p21+ hepatocytes. Dual immunofluorescence staining of p21 (red) and (C) hepatocyte marker HNF4α (green) and (D) p21 (red) and proliferative marker Ki67 (green) from patients with AH, counterstained with DAPI (blue). Scale bar: 100 µm. *p<0.05, ns=non-significant; **FDR<0.01; ***FDR<0.001; ****FDR<0.0001. AH, alcohol-associated hepatitis; BEC, biliary epithelial cell; DAPI, 4′,6-diamidino-2-phenylindole; FDR, false discovery rate.

Immunohistochemistry confirmed sections from human AH livers had increased *p21* expression, compared with normal and cirrhotic livers (figure 4A); quantification revealed a significantly higher percentage of p21+ hepatocytes in AH, compared with normal and cirrhotic livers. Quantification also revealed more proliferative *Ki67*+ hepatocytes in patients with AH compared with cirrhotic livers (figure 4A). We also observed persistent γH2A.X, a marker of DNA damage response associated with senescence onset, in hepatocytes from patients with AH (figure 4A). While p21 expression was observed primarily in hepatocytes, we also detected p21-positive BEC and inflammatory cells (figure 4B). Additional dual immunofluorescence stainings for both p21/HNF4α and p21/Ki67 confirmed expression of p21 in hepatocytes (figure 4C, online supplemental figure S11A) and highlighted that p21+ cells did not coexpress Ki67, confirming that they were not in a proliferative state and likely represent cell cycle arrest and senescence (figure 4D, online supplemental figure S11B).

Reanalysis of other AH datasets confirmed these results. Microarray data^26^ revealed that senescence markers were significantly upregulated in AH versus normal liver (online supplemental figure S12A); hepatocyte markers were significantly downregulated (online supplemental figure S12B). Reanalysis of RNA-seq data^27^ similarly showed that senescence markers were significantly upregulated in sAH versus normal liver (online supplemental figure S12C); again, hepatocyte markers were significantly downregulated (online supplemental figure S12D). Analysis of publicly available AH data^25 27^ also suggests that the upregulation of protein complex subunits identified in ISAIAH data with decreasing MELD is a reversal of a downregulation with progressing ALD, with many COX (online supplemental figure S12E,F) and proteasomal subunits (online supplemental figure S12G,H) significantly downregulated between normal liver and AH.

These data show that ALD progression towards AH is characterised transcriptomically in human liver by loss of hepatocyte marker expression and dysregulation of the proliferation/senescence axis, from the earliest stage. Our analysis suggests that, following an early proliferative phase during which there is an initial senescent response, disease progression towards AH is characterised by a pervasive senescent phenotype.

### Ethanol-induced toxicity in VL-17A cells directly increases senescence marker expression in vitro

**Figure 5:**
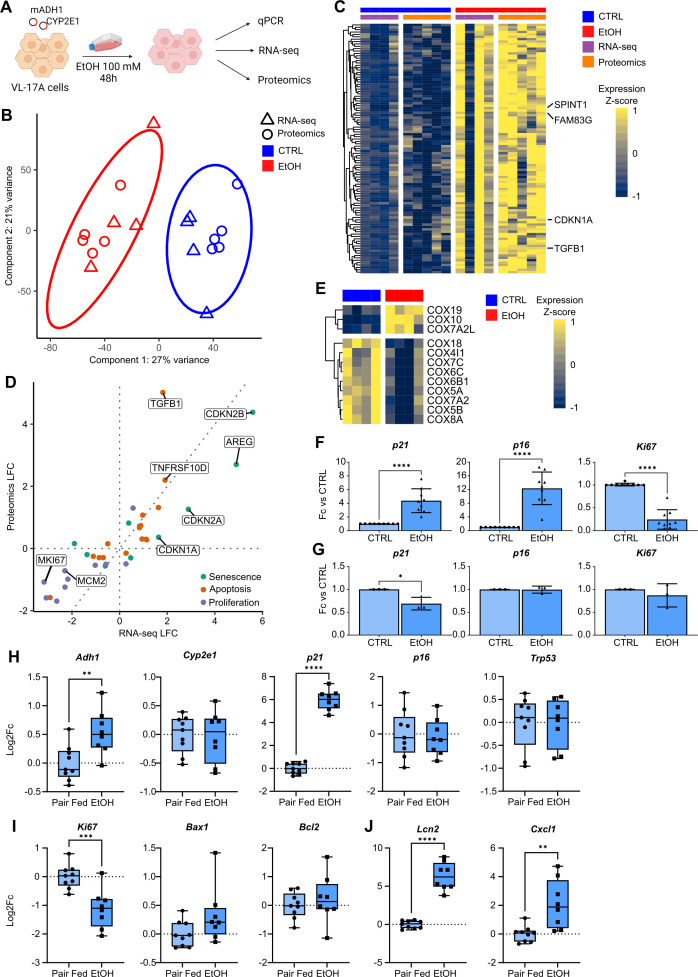
Ethanol-induced toxicity in the hepatocyte VL-17A cell line directly induces senescence marker expression in vitro. (A) Schematic of in vitro experimental design. (B) Scatterplot of RNA-seq and proteomics samples in dimensionally reduced space. (C) Heatmap of progressive ALD markers also significantly upregulated in VL-17A cells. (D) Fold change in RNA-seq and proteomic analysis for significantly DE senescence, apoptosis and proliferation markers. (E) Heatmap of COX subunits significantly DE in RNA-seq of VL-17A cells. (F) Gene expression changes in ethanol-treated (EtOH; n=9) versus untreated VL-17A cells (CTRL; n=9) (mean±SD). (G) Differences in expression in senescence and proliferation markers between CTRL (n=3) and EtOH (n=3) HepG2 cells (mean±SD). Gene expression changes in (H) ethanol-metabolising enzyme *Adh1,* senescence markers (*p21*, *p16* and *Trp53*), (I) proliferation marker *Ki67* and (J) SASP/inflammation markers (*Cxcl1* and *Lcn2*) between pair-fed (n=9) and ethanol-fed (n=8) mice. *p<0.05; **p<0.01; ***p<0.001 ****p<0.0001. ALD, alcohol-related liver disease; COX, cytochrome c oxidase; DE, differentially expressed; Fc, fold change; SASP, senescence-associated secretory phenotype.

To test if treating hepatic cells in vitro directly with ethanol (EtOH) recapitulates the senescence response observed in patients with AH, we used VL-17A cells, a modified human hepatocellular HepG2 cell line.^16^ Cells were treated with 100 mM EtOH for 48 hours^16^ before collection for RNA-seq and mass spectrometry (figure 5A).

Multiomic dimensionality reduction showed samples separated with most variance (component 1; 27%) due to EtOH treatment in both RNA-seq and proteomics (figure 5B). Overenrichment analysis of genomic features strongly correlated (>=90%) with component 1 indicated significant enrichment of p53 pathway genes (online supplemental figure S13A). Features with a strong positive correlation include MKI67, indicative of increased proliferative potential in untreated cells. Apoptosis-associated genes were also strongly correlated (online supplemental figure S13B).

Integrative GSEA revealed enrichment of the P53 pathway and apoptosis; gene set members significantly DE at both mRNA and protein level showed strong concordance between data modalities (online supplemental figure S13C,D). Features significantly upregulated at both mRNA and protein level included those progressively upregulated with worsening AH (online supplemental figure S9A), including CDKN1A (figure 5C). Our multiomic data recapitulated features of AH observed above, including hepatocyte marker downregulation and enrichment of the inflammatory response and hypoxia gene sets, driven by upregulation in EtOH-treated cells (online supplemental figure S13E,F).

72% of significantly DE proteins were also significantly DE in RNA-seq (online supplemental figure S13G). Senescence, apoptosis and proliferation features significantly DE in both modalities were consistent in their expression changes. As above, senescence- and apoptosis-related features were upregulated in EtOH-treated cells; proliferation markers were downregulated (figure 5D). Leveraging the increased resolution of RNA-seq revealed that the most significantly DE COX subunits were downregulated in EtOH-treated cells (figure 5E), as in ISAIAH data. GSEA identified bile acid and fatty acid metabolism among processes most significantly enriched.

To investigate how our in vitro model recapitulated human data, we compared our data to InTEAM data.^25^ Despite different cellular contexts, we found a modest yet significant positive correlation in log fold change across all genes, comparing our model to the AH versus normal comparison in InTEAM data (online supplemental figure S14A). A similar correlation was observed among genes significantly DE in at least one of these datasets (online supplemental figure S14B). 57% of all genes were consistent in their direction of dysregulation. Given the lack of cellular context in our in vitro model, we also compared for a set of high-confidence hepatocyte markers computed from the LCA^20^ and again found a significant correlation, with 62% consistent in their direction of dysregulation (online supplemental figure S14C), the majority downregulated following EtOH injury. We performed similar correlation tests for panels of senescence-, apoptosis- and proliferation-related genes (online supplemental figure S14D–F). While most significantly DE senescence- and apoptosis-related genes were consistent in their expression between VL-17A cells and InTEAM livers, there were some exceptions, such as TP53, significantly decreased in EtOH-treated cells but increased in AH livers versus normal. Many proliferation and cell cycle markers (online supplemental figure S14F) were increased in AH livers but decreased in EtOH-treated cells, notably MKI67, cell cycle regulators CDC25A and CDC25B, and the E2F activators E2F1 and E2F2 (online supplemental tables 3–6).

Real-time quantitative polymerase chain reaction (qPCR) confirmed our proteomics data; alcohol-metabolising enzymes as well as *p21* and *p16* were significantly upregulated in EtOH-treated VL-17A cells versus untreated, while *Ki67* decreased (figure 5F, online supplemental figure S15A). *BAX*, an apoptotic marker, was significantly decreased; antiapoptotic marker *BCL2L1* was increased, suggesting that cells were directed to a senescent, rather than apoptotic, state after 48 hours of EtOH exposure (online supplemental figure S15B).

When the HepG2 parental cell line was treated with EtOH under the same conditions (online supplemental figure S15C), no *CYP2E1* expression was observed; *ADH1A* and aldehyde dehydrogenase 2 (*ALDH2*) had very low expression (online supplemental figure S15D). No significant changes were observed in proliferation (*Ki67*) or senescence (*p16*); *p21* significantly reduced its expression after EtOH treatment (figure 5G). These results indicate that ethanol metabolism is required for the induction of hepatocyte senescence following acute exposure.

To confirm our VL-17A results, we treated isolated primary mouse hepatocytes with EtOH (online supplemental figure S16A). Previous studies demonstrated the link between ethanol exposure and induction of cell death in hepatocytes in vitro.^28^ Similar results were observed: *Cyp2e1* expression significantly increased, while the expression of apoptotic (*Bax*) and antiapoptotic genes (*Bcl-2*) did not significantly change following 48 hours of EtOH treatment (online supplemental figure S16B). We found a trend to increase *Adh1* and *Aldh2*, but did not observe significant changes in *p21* expression (online supplemental figure S16B). *p16* and *p53* were downregulated after EtOH treatment. Expression of EtOH-metabolising enzymes rapidly decreased over time (data not shown), potentially explaining the lack of a senescence response in these cells when metabolisation capacity is decreased, as observed in HepG2 cells.

One histological characteristic of AH is biliary cell expansion (ductular reaction).^7^ To assess the effects of EtOH on proliferation and senescence induction in BEC in vitro, we treated BEC obtained from human liver organoids (online supplemental figure S16C). No significant differences in the expression of ADH1, ALDH2 or senescence, proliferation or BEC markers were observed in EtOH-treated versus untreated cells (online supplemental figure S16D), suggesting that BECs are resistant to the senescence-inducing effects of alcohol in vitro. Longer exposure to ethanol or paracrine signals released by hepatocytes or other hepatic cell populations may be required to induce proliferation and senescence in BEC, as observed in AH.

Together, these data show that ethanol-induced injury directly induces hepatocyte senescence in an in vitro model, at both mRNA and protein level, and ethanol metabolism may be required to induce senescence.

### p21 expression is increased only at transcriptomic level after chronic ethanol feeding in mice

Given the direct effect of ethanol observed in vitro, we next studied changes in senescence markers in the NIAAA mouse model of chronic plus binge ethanol feeding.^29^ PCR showed increased expression of ethanol-metabolising enzyme *Adh1*together with *p21* in ethanol-fed mice compared with controls. However, no differences were observed in other senescence markers (eg, *p16* and *p53*) as well as *Bax* and *Bcl-2* (figure 5H,I). *Ki67* was significantly downregulated in EtOH-treated mice, as observed in vitro (figure 5I). EtOH feeding also induced the expression of SASP/inflammatory markers (eg, *Lcn2*, *Cxcl1*) (figure 5J).

Assessment of Ki67 and p21 protein-level expression in this model showed isolated/sporadic p21+ hepatocytes in both groups (data not shown); EtOH-induced damage in this model was mild and insufficient to drive a senescence response at protein level; this model likely better represents early ALD.

## Discussion

Excessive alcohol consumption kills around 3 million people (5.3% of all deaths) worldwide annually.^30^ Transcriptomic data are vital in identifying potential therapeutic targets for AH. However, no studies have leveraged paired biopsies to assess transcriptional changes following partial recovery from an acute event in an underlying chronic condition. Here, we provide the first such analysis, using data from the ISAIAH trial^5^ to identify elements involved in alcohol-related injury and resolution.

Our longitudinal data revealed a downregulation of senescence markers in liver with AH resolution following peak injury. Further bioinformatics analysis identified a transcriptomic signature of senescence, apoptosis and proliferation that is increasingly dysregulated with worsening ALD with senescence markers significantly upregulated in AH. While ISAIAH transcriptomic data suggested that this dysregulation is partially reversed with AH resolution, we observed similar levels of senescence markers after 28 days at protein level. Persistent presence of senescence cells may contribute to diminished/slower regeneration, prolonged fibrosis and inflammation through paracrine mechanisms.^13 31^ Altogether, this may partially explain the poor liver regeneration and functional improvement rates in AH, even following temporary alcohol cessation, as liver restoration may take many months.^32^ While transcriptional changes are apparent after 28 days, we anticipate that the extensive changes we observed at this time point could potentially continue over an extended period.

Cellular senescence is a key response to persistent hepatic injury in chronic and acute severe liver disease,^11^ preventing cell death. In AH, mechanisms of both chronic and acute induction of senescence are likely present. Senescence itself is a disease mechanism, enhancing fibrosis, triggering inflammation and impairing regeneration^11^; whether senescence onset in AH is solely a consequence of, or also a contributing factor to, liver injury is unknown. Senescent cells impact the microenvironment by releasing SASP factors, eliciting a neighbouring senescence response^11^ and immune cell recruitment. Senescent cells thus contribute to driving pathology, as well as resulting from injury. We observed higher expression of SASP factors in AH; however, our data preclude us from definitively stating whether this originates from senescent or non-senescent (eg, inflamed or fibrotic) cells contributing to the underlying inflammatory state of AH.^33^

Senescent hepatocytes have been correlated with disease severity in MASLD,^34^ and with increased adverse outcomes and fibrosis stage in MASLD and ALD.^10 35^ Senescence may, therefore, contribute to ALD progression; however, other cellular responses (eg, inflammation and fibrosis) also induce liver injury. We identified two senescence-related complexes, COX and the proteasome, dysregulation of which may act as senescence-induction mechanisms in ALD/AH.

Ethanol directly affects mitochondrial function and integrity^36^; COX is a direct target of ethanol metabolisation in the mitochondrial compartment,^37^ with reduced expression following chronic alcohol feeding in rodents.^38^ During early ALD, dysfunctional mitochondria release COX factors, exacerbating an inflammatory and apoptotic response.^39^ As increased ROS is in turn a hallmark of senescence, we hypothesise that COX reduction may be involved in ALD senescence onset by controlling levels of factors such as p21, p16 or p53 in a feedback loop. Mitochondria are dysfunctional in senescent cells; conversely, the induction of senescence in hepatocytes leads to mitochondrial dysfunction.^34^ How the cell’s microenvironment guides it towards senescence or apoptosis during ALD progression requires further investigation but may involve ‘pivotal factors’ at the crossroads between these processes, such as DCR2.^23^

Proteasome activity is affected by CYP2E1 induction through alcohol metabolism, again via ROS generation.^40^ This functional impairment contributes to alcohol-induced steatosis and liver injury, leading to hepatic protein accumulation in the form of Mallory–Denk Bodies, which are associated with senescence.^41^,^43^ During replicative senescence, proteasome function is impaired; its inhibition has also been shown to induce a senescent phenotype in primary human fibroblast cultures.^44^ We found several proteasomal subunits downregulated in AH; however, whether ethanol-directed proteasome degradation induces a senescent phenotype in hepatocytes remains to be shown.

We found no significant transcriptomic differences associated with Canakinumab (anti-IL-1β) in ISAIAH data. While preclinical studies in mice highlighted the importance of the IL-1β signalling pathway in ALD pathogenesis,^45^ clinical trials targeting either IL-1β^5^ or blocking the IL-1 receptor with IL-1Ra have shown no reduction in the inflammatory response in patients with AH.^46 47^ IL-1β also has a known role in SASP response; therefore, blocking via Canakinumab may affect SASP. Since SASP encompasses many other factors, a future therapeutic strategy may be a wider alteration of the senescence environment, potentially via the SASP response or limiting senescent cells with senomorphic or senolytic drugs. However, the impact of modulating senescent cells in ALD is unknown.

Recently, it has been reported that ethanol exposure to primary hepatocytes increases senescence markers p21 and p53.^48^ Our multiomic analysis provides a deeper insight into the hepatocyte phenotype following ethanol treatment in vitro. Ethanol induced senescence at transcriptomic level; however, a complete senescence phenotype was not observed at protein level. This partial phenotype may be explained by the acute treatment compared with chronic injury in AH, and the lack of a wider cellular context, in which other cell types may interact with hepatocytes, exacerbating a senescence response. Ethanol treatment did not induce proliferation or senescence markers in BEC, suggesting that its effect on ductular cell expansion is indirect and likely related to hepatocyte senescence.^49^

The lack of suitable validatory models is a limitation; current animal models insufficiently capture the full spectrum of human AH. Further, although our in vitro model recapitulates the senescent response observed in patients with AH, the lack of cellular context precludes us from investigating potential mechanisms, such as the SASP response observed in ISAIAH data, or paracrine senescence between cell types. While we identified dysregulation of senescence-related complexes with disease severity that may explain elevated ROS production, further experimental work is required to elucidate the cell types and mechanisms involved, including single-cell, spatial multiomics and/or exhaustive immunocytochemistry analysis on AH liver biopsies, which may lead to a more conclusive characterisation of the senescence phenotype in hepatocytes following ethanol insult. Finally, MELD may underestimate AH severity in women and malnourished patients.^50^

In this study, we show, for the first time, a reduced senescence response in liver following partial resolution of an acute episode of ethanol-induced injury, using a novel longitudinal transcriptomic dataset. We further identified senescence and associated pathways as involved in ALD progression, specifically during AH, correlating with disease severity. Our in vitro data indicate that hepatocyte senescence is a direct effect of ethanol. Future therapies might consider targeting senescence or associated pathways in early ALD to limit the severity of acute episodes.

## Supplementary material

10.1136/gutjnl-2024-334094 online supplemental file 1

## Data Availability

Data are available in a public, open-access repository.
