# Supplementary material for: Longitudinal paired liver biopsies and transcriptome profiling in alcohol-associated hepatitis reveal dynamic changes in cellular senescence
Source: Gut. Author manuscript; Available in PMC 2025 Aug 8. (PMC12331437; doi:10.1136/gutjnl-2024-334094)
Supplement: Supplementary file 1 [file NIHMS2073372-supplement-supplemental_material.pdf]

Supplementary material

Longitudinal paired liver biopsies and transcriptome profiling in alcohol-associated hepatitis reveal dynamic changes in cellular senescence

Authors

Daniel Rodrigo-Torres[1] and Alastair M. Kilpatrick[1,2], Sofia Ferreira-Gonzalez[1,3], Rhona E. Aird[1], Stephen Rahul Atkinson[2], Victoria L. Gadd[1], Tak Yung Man[1], Luke D. Tyson[2], Gopal Krishna R. Dhondalay[2,4], Nikhil Vergis[2], Gavin E. Arteel[5,6], Mark R. Thursz[2], Laura Martinez-Gili[2,4] and Stuart J. Forbes[1,*]

Table of Contents

Supplementary Methods 3

In vitro studies 3

Primary mouse hepatocytes 3

Biliary epithelial cells 4

Mouse model of chronic plus binge ethanol feeding 5

Human tissue 6

Image analysis 6

p21 and p53 quantification in ISAIAH biopsies 6

Ki67 and p21 quantification in liver tissue 7

VL-17A cells RNA sequencing 7

RNA-seq bioinformatics preprocessing 8

ISAIAH RNA-seq quality control 9

RNA-seq differential expression analysis 9

ISAIAH RNA-seq statistical modelling 10

VL-17A proteomics 11

Functional analysis of omics data 12

VL-17A multiomic analysis 12

Rodrigo-Torres D, *et al. Gut* 2025;0:1–14. doi: 10.1136/gutjnl-2024-334094

Affymetrix array analysis 13
Liver Cell Atlas scRNA-seq data 13
AH scRNA-seq data 14
Bulk RNA-seq deconvolution 14
Supplementary Figure captions *16*
Supplementary Fig. 1: Senescence and apoptosis pathways are downregulated following AH resolution. 17
Supplementary Fig. 2: Inflammatory response and cell type markers are modulated following AH resolution. 19
Supplementary Fig. 3: Proportions of hepatocyte cells and hepatocyte marker expression are predicted to increase following AH resolution 21
Supplementary Fig. 4: Hypoxia and ciliary pathways are enriched following AH resolution. 22
Supplementary Fig. 5: AH resolution reverses ALD-related proliferation and metabolism dysregulation. 25
Supplementary Fig. 6: Hepatic and inflammatory dysregulation is reversed following AH resolution. 27
Supplementary Fig. 7: Senescence-related processes are disrupted by COX subunit dysregulation in AH. 29
Supplementary Fig. 8: Senescence-related processes are disrupted by proteasomal dysregulation in AH. 31
Supplementary Fig. 9: A senescence signature is enriched in genes progressively upregulated with worsening ALD. 33
Supplementary Fig. 10: GSEA of AH vs normal liver reveals senescence and metabolic dysregulation. 35
Supplementary Fig. 11: Single channels for Figure 4C and 4D in AH patients. 37
Supplementary Fig. 12: Senescence and related protein complex subunits are dysregulated in AH. 39
Supplementary Fig. 13: Ethanol induces a senescent response in the hepatocyte VL-17A cell line in multiomic analysis. 41
Supplementary Fig. 14: Fold change direction between whole-liver and hepatocyte (VL-17A) cells is largely consistent. 43
Supplementary Fig. 15: Ethanol metabolism is required for hepatocyte senescence induction in a hepatocyte (VL-17A) cell line. 45
Supplementary Fig. 16: Effect of ethanol in other systems. 47
Supplementary Tables *48*
Supplementary Table 1. List of antibodies for immunostaining 48
Supplementary Table 2. Primers for RT-qPCR (Qiagen QuantiTect Primer Assay). 49
Supplementary Table 3. Differential expression of senescence markers in different datasets. 50
Supplementary Table 4. Differential expression of apoptosis markers in different datasets. 50
Supplementary Table 5. Differential expression of proliferation markers in different datasets. 52

Supplementary Table 6. Differential expression of hepatocyte markers in different datasets. 53
Supplementary References... 54

Supplementary Methods

In vitro studies

HepG2 cells (ATCC HB-8065) were cultured in 25 cm^2 flasks in RPMI 1640 Medium (Thermo Fisher Scientific) supplemented with 10% FCS and 1% L-glutamine (Invitrogen) in the presence or absence of ethanol at a concentration of 100 mM for 48 hours. Cells were collected in RLT buffer for RNA extraction.

Primary mouse hepatocytes

Experiments involving animals were conducted under ARRIVE guidelines, as well as procedural guidelines, severity protocols and with ethical permission from the University of Edinburgh Animal Welfare and Ethical Review Body and the UK Home Office. Animals were housed in IVC cages under a 12-hour light/dark cycle and allowed access to food and water ad libitum.
For primary mouse hepatocytes experiments, cells were isolated from 8-10 week C57BL/6 male and female mice (n=4). Briefly, mice under terminal dose of anesthesia were cannulated through the portal vein and perfused with 50 mL Liver Perfusion Medium (Gibco) followed by 50 mL Liver Digest Medium (Gibco) pre-warmed to 37°C. The liver was excised before hepatocytes were mechanically disassociated and filtered through a 70 µm filter (Corning). Hepatocytes were centrifuged twice at 50g for 2 minutes with break off and resuspended in Williams E Media, before purification with a 35% Percoll density gradient (Sigma) at 500g for 10

minutes with break off. Viable hepatocytes were pelleted and resuspended in Williams E Media after removing Percoll, then centrifuged again at 50g for 2 minutes before being plated at the desired cell density.

Hepatocytes were cultured in p25 flasks (Corning) coated with 1mg/ml of Collagen, Type I solution (Sigma-Aldrich) at a cell density of 80,000-120,000 hepatocytes/cm^2. Hepatocytes were plated in hepatocyte media: Williams E medium (Thermo Fisher Scientific) supplemented with 10% FCS, 1% L-glutamine, 1% Penicillin/Streptomycin and 100 µg/ml primocin (Invivogen) overnight. The next day, cells were washed with Williams E medium; hepatocyte media with no FCS was added to the cells and incubated in the presence/absence of 150 mM ethanol for 48 hours[1]. Cells were collected in RLT buffer for RNA extraction at the end of the experiment.

Biliary epithelial cells

For biliary epithelial cells experiments, human BEC grown as 3D liver organoids conditions[2] and derived from untransplantable human livers were dissociated mechanically and enzymatically as follows: organoids were washed twice with cold PE (PBS + 0.5 M EDTA (Invitrogen)) and incubated at 37°C with 1 Unit of Dispase (Gibco) for 30 minutes. Once they were detached from the plate, organoids were transferred to a 15 ml tube and centrifuged at 300g for 5 minutes. Supernatant was carefully removed and a 2.5ml mixed solution of 10x and 1x TrypLE (Gibco) (1:5 ratio) was added to the cells. Organoids were pipetted up and down before incubating them for 20 minutes in a water bath at 37°C. Once organoids were disaggregated in singlets, tubes were filled with Advanced DMEM/F12 media (Gibco) and centrifuged at 300g for 5 minutes. Supernatant was removed, cells resuspended in BEC media and counted to plate them at the desired density ($7.5x10^4$ cells/cm^2) in a 6 well plate. Once cells reached around 80% confluency, fresh organoid culture

media (as described in [2]) and 150 mM ethanol were added to the cells for 48 hours. Experiments were performed in technical duplicates and two different organoid lines were used.

In all *in vitro* experiments, media and ethanol were replaced every 24h and flasks/dishes were parafilm wrapped to minimise ethanol loss due to evaporation.

Mouse model of chronic plus binge ethanol feeding

Male C57BL/6J mice (8 weeks old) were purchased from Jackson Laboratory (Bar Harbor, ME) and housed in a pathogen-free barrier facility by the Association for Assessment and Accreditation of Laboratory Animal Care. All procedures were approved by the local Institutional Animal Care and Use Committee (University of Pittsburgh, USA). Animal were allowed standard laboratory chow and water ad libitum. Sample sizes were selected taking into consideration similar previous experiments, the model and the injury systems. Mice (n=17) were randomly separated into two groups and subjected to different diet regimes in separate cages*:* the first group received ad libitum 5% *ethanol in a* liquid *Lieber de-Carli diet (Bio-Serv) for 10 days* (n=8). The second group received an isocaloric control diet *(Bio-Serv)* (n=9). On day 11, mice were orally gavaged with ethanol (5 g/kg body weight) or isocaloric maltose dextrin (9 g/kg body weight) respectively. After 9 hours, the mice were anesthetized with ketamine/xylazine. Blood was collected from the vena cava just prior to sacrifice by exsanguination and citrated plasma was stored at -80°C for further analysis. Portions of liver tissue were frozen immediately in liquid nitrogen for PCR, and RNA was extracted using RNA Stat60 (Tel-Test, Ambion, Austin, TX) and chloroform for subsequent PCR analysis. Other portions were fixed

in 10% neutral buffered formalin or embedded in frozen specimen medium (Tissue-Tek OCT compound, Sakura Finetek, Torrance, CA) for subsequent sectioning and immunohistochemistry analysis. No animals were excluded during the experiment or during the data analysis.

Human tissue

Normal human liver samples were collected and embedded in paraffin for immunohistochemistry analysis from livers discarded for transplantation. Ethical approval for the use of these tissues was obtained from the NHS Lothian research ethics committee (reference number 15/SS/0218), NHS Lothian Research and Development (Project No. 2015/0408), and the NHS blood and transplant (NHSBT) ethics committee (Research Innovation and Novel Technologies Advisory Group (RINTAG) (registered as study 56)).

Paraffin-embedded liver biopsy samples of patients with AH (n=5), and liver explants from patients with alcohol induced cirrhosis (n=5) were collected by Lothian NRS Bioresource (REC reference number 15/ES/0094). All samples were selected based on histology assessment and aetiology confirmation by an expert pathologist.

Image analysis

Images were acquired using a Nikon Eclipse e600 microscope (Nikon) at up to 40x magnification using Micropublisher 6 CCD camera and Q-Imaging ImagePro premier software (Teledyne Photometrics, USA). Images were analysed using Fiji software.

p21 and p53 quantification in ISAIAH biopsies

For p21 staining, 5-10 non-overlapping pictures per biopsy were randomly taken at 40x magnification in a subset of ISAIAH patients randomly selected based on tissue availability (n=12 paired biopsies). For p53 staining, 8-20 non-overlapping pictures per biopsy in n=7 paired randomly selected biopsies from a subset of ISAIAH patients were randomly taken at 40x magnification. Number of *p21+ and p53*+ hepatocytes were manually counted; quantification is presented as percentage of positive hepatocytes.

Ki67 and p21 quantification in liver tissue

At least 10 non-overlapping pictures were randomly taken at 40x magnification from normal livers' tissue discarded for transplantation (Norm) (n=5-6), liver biopsies from AH patients (AH) (n=4) and cirrhotic explants (Cirr) (n=5). Number of Ki67+ and p21+ hepatocytes were manually counted; quantification is presented as percentage of positive hepatocytes.

VL-17A cells RNA sequencing

Libraries were prepared from 500ng of each total-RNA sample using the NEBNext Ultra II Directional RNA Library Prep kit (NEB #7760) and the Poly(A) mRNA magnetic isolation module (NEB #E7490) according to the provided protocol. Poly(A) containing mRNA molecules were purified using poly-T oligo attached magnetic beads. Following purification, the mRNA was fragmented using divalent cations under elevated temperature and primed with random hexamers. Primed RNA fragments were reverse transcribed into first strand cDNA using reverse transcriptase and random primers. RNA templates were removed, and a replacement strand synthesised incorporating dUTP in place of dTTP to generate ds

cDNA. AMPure XP beads (Beckman Coulter, #A63881) were used to separate the ds cDNA from the second strand reaction mix, providing blunt-ended cDNA. Multiple indexing adapters were then ligated to the ends of the ds cDNA to prepare them for hybridisation onto a flow cell, before 10 cycles of PCR were used to selectively enrich DNA fragments that had adapter molecules on both ends and amplify the amount of DNA in the library suitable for sequencing. Libraries were purified using AMPure XP beads, quantified by fluorometry using the Qubit dsDNA HS assay and assessed for quality and fragment size using the Agilent Bioanalyser with DNA HS Kit (#5067-4626). Fragment size and quantity measurements were used to calculate molarity. Sequencing was performed on an Illumina NextSeq 2000 platform (Illumina Inc, #SY-415-1002) using the NextSeq 1000/2000 P3 Reagents (200 cycles) v3 Kit (#20040560). Libraries were combined in an equimolar pool and run over a single P3 flow cell at the Edinburgh Clinical Research Facility (ECRF), Western General Hospital, Edinburgh, UK.

RNA-seq bioinformatics preprocessing

RNA-seq data was subject to a common bioinformatics preprocessing pipeline, with minor variation as described.

Raw RNA-seq data from the InTEAM consortium[4] was downloaded from dbGaP (phs001807.v1.p1). Raw RNA-seq data from Hyun, et al., was downloaded from NCBI Gene Expression Omnibus (GEO) (GSE143318)[5]. ISAIAH and VL-17A RNA-seq data were generated as described above.

Read quality was initially assessed (per lane, as required) using FastQC (v.0.11.9) and MultiQC (v.1.11). Illumina standard adapter sequences were trimmed using Cutadapt (v.1.16)[6]; low quality bases (Phred<20) were also trimmed. For ISAIAH

RNA-seq data, we additionally trimmed 3bp (deriving from the Pico V2 smart adapter) from the 5' end of R2 reads as recommended in the SmarterV2 protocol. Subsequent quality assessment confirmed adapter contamination of <0.1% per sample in all datasets. Following merging of sequencing lanes as required, sequence reads were aligned to the human reference genome (GRCh38) with STAR (v.2.7.1a)[7]**.** Aligned reads were quantified using RSEM (v.1.2.28)[8] and data imported into R for downstream analysis.

ISAIAH RNA-seq quality control

Outlying samples from the ISAIAH RNA-seq dataset were identified by performing principal component analysis on the complete data using the ropls Bioconductor package (v.1.30)[9]**.** We identified and removed n=6 outlying samples based on score distance and orthogonal distance in the PCA observation diagnostics, retaining n=59 samples for downstream analysis. Additional analysis of patient data confirmed that these outliers were due only to technical effects. Following sample filtering, 27 ISAIAH patients had matched (d0 and d28) samples (n=54). 3 patients only had a d0 sample and 2 patients only had a d28 sample; data from these patients was retained, to use as much data as possible for differential expression analysis.

RNA-seq differential expression analysis

The InTEAM cohort contains RNA-seq data from patients at progressing ALD stages, including normal liver (n=10), early alcoholic steatohepatitis (n=12) and severe AH (n=18)[4]. Data from Hyun, et al.[5]**,** contains RNA-seq of alcoholic hepatitis patients

(n=5) and healthy controls (n=5). The VL-17A data generated in this study contains cells exposed to ethanol as described above (n=4) and control cells (n=4). For each dataset, genomic features were filtered using the filterByExpr function in the edgeR Bioconductor package[10], using the default parameter settings and grouping samples by treatment or disease group, as appropriate. Apart from ISAIAH RNA-seq data (see in Supplementary information), differential expression analysis was computed between groups as appropriate using DESeq2 (v.1.26.0)[11]. P-values were corrected for multiple hypothesis testing using the false discovery rate (FDR) method. Genes with FDR<0.05 were considered significantly differentially expressed.

For visualization of RNA-seq data, read counts were normalized with respect to library size using the regularized log (rlog) transform[11].

ISAIAH RNA-seq statistical modelling

Differential expression for ISAIAH data was computed using three linear mixed-effects (LME) models[12], implemented using the lme4 R package:

1) feature ~ **time:treatment** + time + age + sex + (1|subject_id)
2) feature ~ **time** + age + sex + (1|subject_id)
3) feature ~ **MELD** + time + age + sex + (1|subject_id)

Model 1 tested for effects of canakinumab treatment with respect to placebo as a time:treatment interaction term[13]. Patient age and sex were covariates, with patient ID as a random effect, to leverage matched samples where possible. Model 2 tested for overall dynamic changes in expression at day 28 with respect to day 0, with 'timepoint' as a categorical term. The same covariates and random effects were used. Model 3 tested for effects with respect to MELD, with MELD as the main model term, patient age, sex and sample timepoint were covariates, again with patient ID

as a random effect. P-values were obtained with the likelihood ratio test, using a nested model without the main variable (indicated in bold) but keep the rest of the covariates. As above, p-values were corrected for multiple hypothesis testing using the false discovery rate (FDR) method. Genes with FDR<0.05 were considered significantly differentially expressed.

For visualization of RNA-seq data, read counts were normalized using the centred log ratio (CLR) transformation. Heatmap visualisation of ISAIAH data used the ComplexHeatmap Bioconductor package (v2.14)[14]. Correlation plots of CLR transformed read counts vs MELD score used the Pearson method to compute correlation coefficients and p-values.

The RNA-seq data generated in this study (ISAIAH; VL-17A) has been deposited in NCBI GEO (SuperSeries GSE270043).

VL-17A proteomics

1 µg of de-salted peptides were loaded onto a 25 cm emitter (Odyssey, IonOptiks, Australia) using a RSLC-nano uHPLC systems connected to a Fusion Lumos mass spectrometer (both Thermo, UK). Peptides were separated by a 70 min linear gradient from 5% to 30% acetonitrile, 0.05% acetic acid. The mass spectrometer was operated in DIA mode, acquiring a MS 350-1650 Da at 120k resolution followed by MS/MS on 45 windows with 0.5 Da overlap (200-2000 Da) at 30k with a NCE setting of 27. The raw data files underwent analysis and quantification utilizing the DIA-NN software using the default settings. A human proteome FASTA file was employed to compare the calculated peptides. For the analysis, the precursor m/z range was defined from 350 to 1650, while the fragment ion m/z range was set from

200 to 2000. Double-pass mode (High Precision) was enabled. Upon completion of the analysis, TSV (tab-separated values) files were generated from the processed data for subsequent analysis and interpretation. Differential expression was computed between treatment groups; proteins with moderated p-value<0.05 were judged to be significantly differentially expressed. The mass spectrometry proteomics data have been deposited to the ProteomeXchange Consortium via the PRIDE partner repository[15] with the dataset identifier PXD053109.

Functional analysis of omics data

Gene set enrichment analysis was computed for the RNA-seq and proteomics datasets using the fgsea Bioconductor package[16] with the MSigDB Hallmark[17], C2 (curated gene sets) and C5 (Gene Ontology subcollection; biological process component) gene sets applied to differential expression results. Gene sets with fewer than 15 genes or more than 500 genes were excluded from analysis; the number of permutations was set to 10,000. Gene sets with FDR<0.05 were judged to be significant.

Additional visualisation of functional data used the pathview Bioconductor package (v1.38)[18]**,** drawing on data from the KEGG database.

VL-17A multiomic analysis

RNA-seq and proteomics data were integrated using the mixOmics Bioconductor package (v.6.22.0)[19]. Prior to integration, data were deduplicated to ensure all gene symbols were valid and unique. We filtered RNA-seq features with the same gene symbol to retain that with the highest mean read count. Proteomics features were filtered similarly, based on highest median intensity across samples. RNA-seq and

proteomics data were then integrated based on gene symbols using the multivariate integrative sparse partial least squares discriminant analysis (MINT sPLS-DA) method in mixOmics. Dimensionality reduction of the integrated dataset was computed on the MINT sPLS-DA model. Integrative analysis of GSEA results was computed using the PathwayMultiomics R package (v.0.0.0.9006)[20]; gene sets with MiniMaxFDR<0.05 were defined as being significantly enriched.

Affymetrix array analysis

Processed Affymetrix Human Genome U133 Plus 2.0 array data was downloaded from NCBI GEO (GSE28619)[21]. The data had been previously subject to RMA background correction, quantile normalization and quality checks. Data was imported into R and samples grouped as healthy controls (*n*=7) or AH patients (*n*=15). Differentially expressed genes were computed using the Limma Bioconductor package (v.3.42.2)[22]. Genes with FDR<0.05 were considered statistically significant.

Liver Cell Atlas scRNA-seq data

We downloaded publicly available scRNA-seq data of human liver cells (NCBI GEO: GSE192742) from the Liver Cell Atlas (LCA)[23] and converted to Seurat format for use in subsequent analysis. To associate significantly DE genes identified in bulk RNA-sequencing with liver cell types, module scores were computed using the AddModuleScore function in the Seurat R package (v4.4)[24] with the LCA scRNA-seq data. Higher scores for each cell-type module reflect higher expression of the input genes. To identify high confidence hepatocyte markers, we computed markers based on LCA cell type annotations and filtered to significantly DE markers expressed in > 80% hepatocytes and < 10% of other cells.

AH scRNA-seq data

We downloaded publicly available scRNA-seq of whole liver from n=5 AH patients (NCBI GEO: GSE255772)[25] and converted to Seurat format for use in subsequent analysis. Cells were annotated using data supplied by the authors; present cell types were: B cells, Basophils, Endothelial cells, Hepatocytes, Monocytes, Macrophages, Neutrophils, NK/NKT cells, pDCs, Proliferating cells, Stellate cells and T cells. Raw scRNA-seq data were merged to a single object and subject to standard scRNA-seq preprocessing (normalisation, scaling and dimensionality reduction) with the Seurat R package. Cells were clustered using the first 30 principal components, with resolution 0.1, as recommended by the authors. A decovolution reference based on this dataset was generated using the IOBR R package (v0.99.0)[26]

Bulk RNA-seq deconvolution

Raw gene expression values from ISAIAH RNA-seq data were transformed to transcript per million (TPM) values using the IOBR R package. This data was deconvolved using the CIBERSORT method [27] within IOBR, specifying 100 permutations for statistical analysis. CIBERSORT provides predicted cell type proportions for each bulk RNA-seq sample. CIBERSORT results were subject to repeated ANOVA with pairwise post-hoc testing as appropriate to determine significant differences in predicted cell type proportions between timepoints; repeated ANOVA allows leveraging paired results for trial participants where available.

Rodrigo-Torres D, *et al. Gut* 2025;0:1–14. doi: 10.1136/gutjnl-2024-334094

Supplementary Figure captions

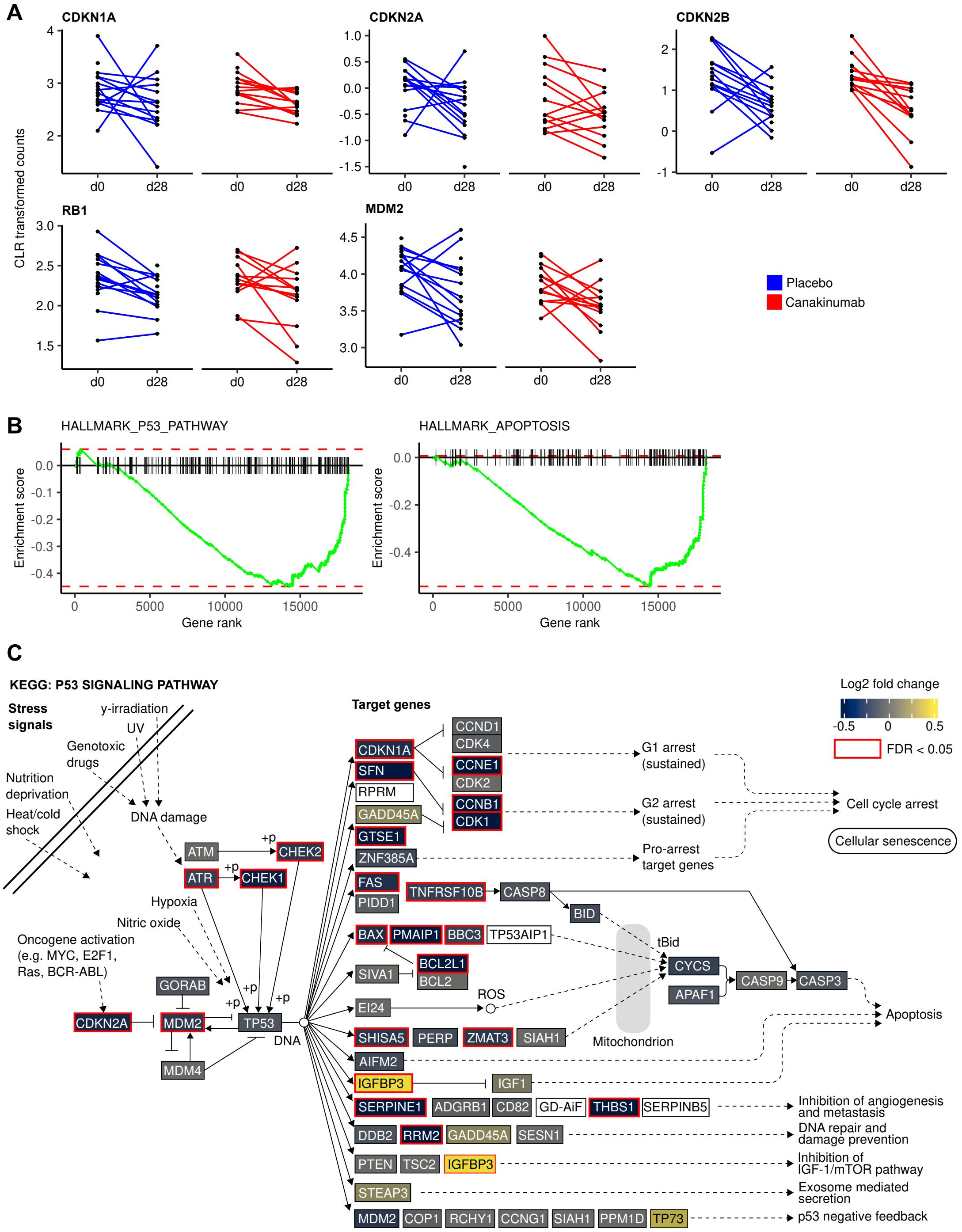

R1 Gut Figure S1

Supplementary Fig. 1: Senescence and apoptosis pathways are downregulated following AH resolution.

(A) CLR transformed read counts for senescence markers at d0 and 28, split by treatment group. (B) GSEA for MSigDB Hallmark P53 pathway and Apoptosis gene sets, comparing d28 and d0 gene expression. (C) KEGG P53 signaling pathway diagram. Pathway members are coloured by coefficient change in d28, vs d0.

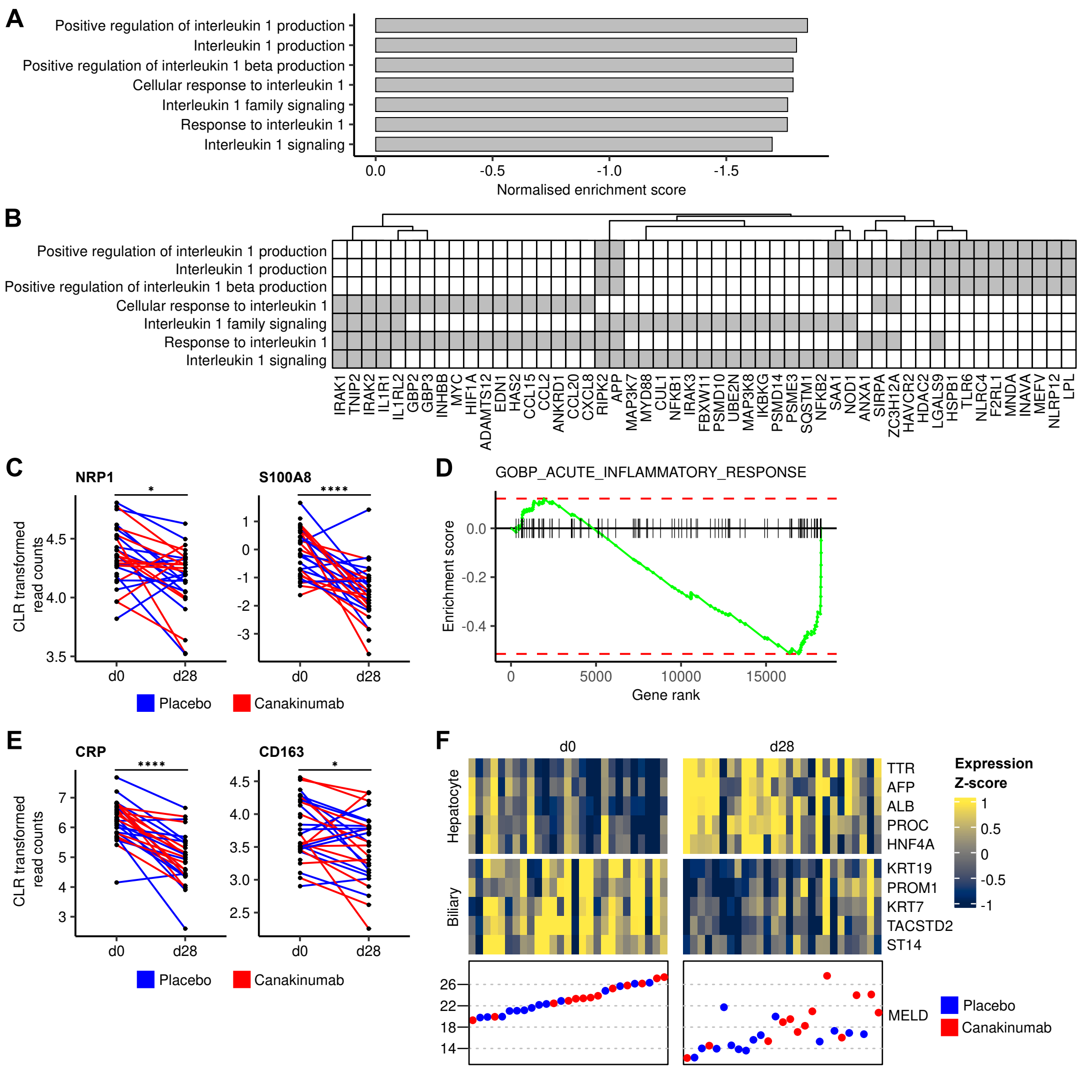

R1 Gut Figure S2

Supplementary Fig. 2: Inflammatory response and cell type markers are modulated following AH resolution.

(A) Normalised enrichment score of IL1 and IL1B-related gene sets. (B) Matrix of genes significantly dysregulated between d0 and d28 and associated with >=2 gene sets identified in A. (C) CLR transformed read counts for pDC markers in ISAIAH patients, at d0, d28. Points represent patients; lines connect matched measurements from the same patient, where available. (D) GSEA for the Gene Ontology Acute Inflammatory Response gene set, comparing d0 and d28 gene expression. (E) CLR transformed read counts for inflammatory markers in ISAIAH patients at d0 and d28. (F) Heatmap of gene expression in ISAIAH patients at d0, d28.

* FDR<0.05; **** FDR<0.0001.

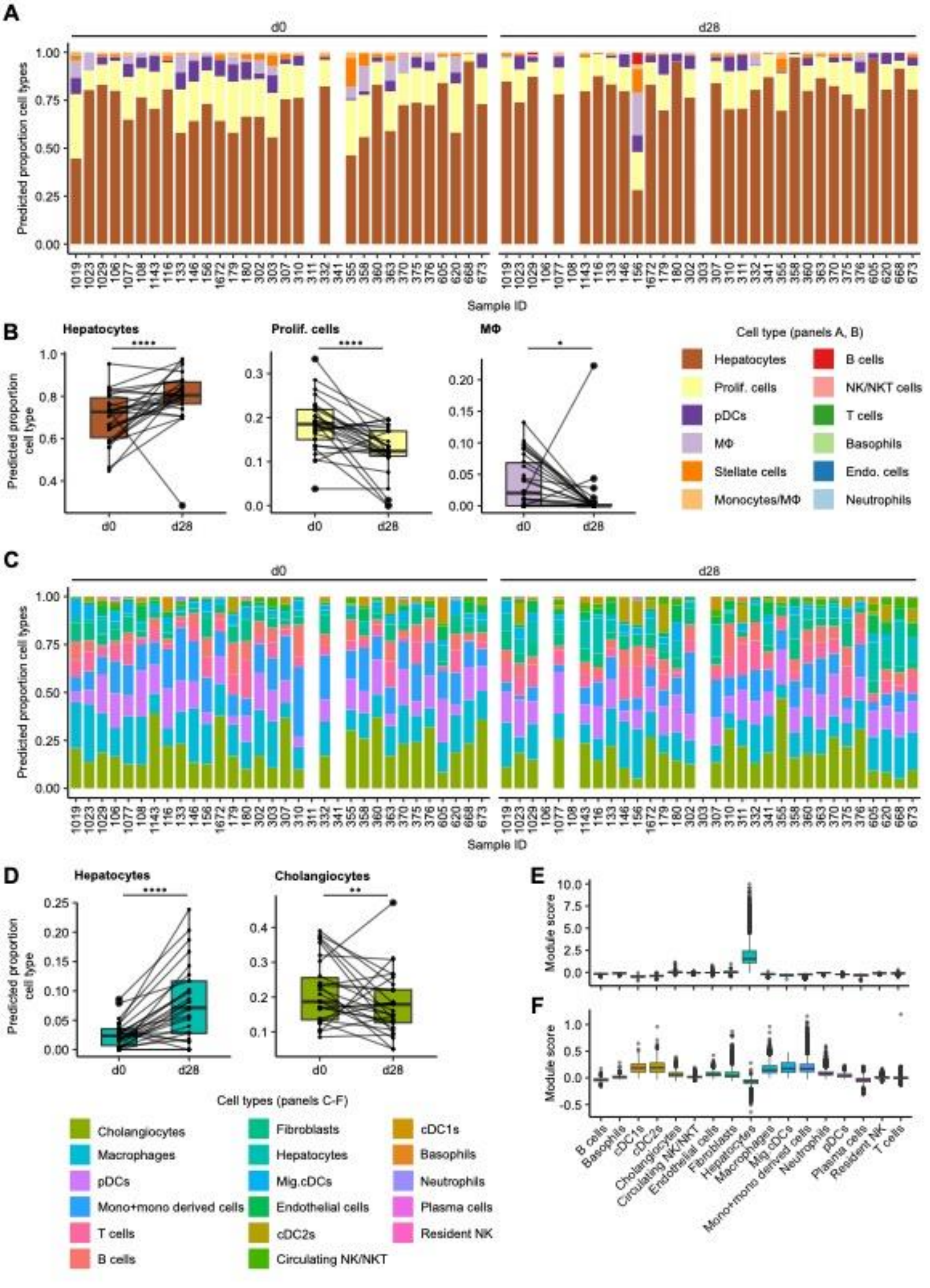

R1 Gut Figure S3

Rodrigo-Torres D, *et al. Gut* 2025;0:1–14. doi: 10.1136/gutjnl-2024-334094

Supplementary Fig. 3: Proportions of hepatocyte cells and hepatocyte marker expression are predicted to increase following AH resolution.

Single-cell RNA-seq (scRNA-seq) aided analysis of ISAIAH bulk RNA-seq data. (A) Predicted proportions of cell types for each ISAIAH bulk-RNA sample, as predicted via deconvolution using CIBERSORT and a scRNA-seq reference dataset of AH liver. (B) Plots of predicted proportions of hepatocytes, proliferating cells (prolif. cells) and macrophages (Mφ) at d0 and d28. Points represent patients; lines connect matched measurements from the same patient, where available. (C) Predicted proportions of cell types as predicted using the Liver Cell Atlas as deconvolutional reference. (D) Plots of predicted proportions of hepatocytes and cholangiocytes at d0 and d28. Points represent patients; lines connect matched measurements from the same patient, where available. Module scores for genes significantly up- (E) and downregulated (F) at d28. * FDR<0.05; ** FDR<0.01; **** FDR<0.0001.

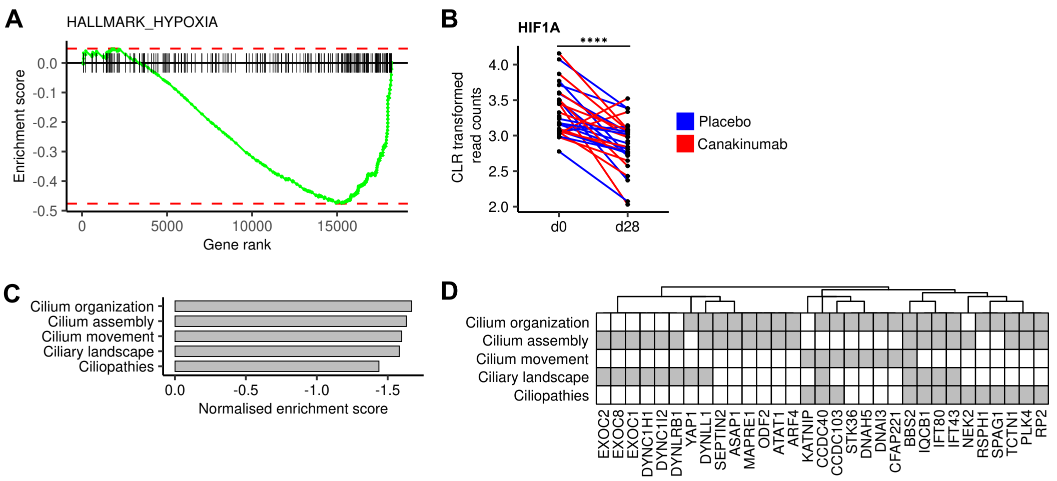

R1 Gut Figure S4

Supplementary Fig. 4: Hypoxia and ciliary pathways are enriched following AH resolution.

Rodrigo-Torres D, *et al. Gut* 2025;0:1–14. doi: 10.1136/gutjnl-2024-334094

(A) GSEA for the MSigDB Hallmark Hypoxia gene set, comparing d28 and d0 gene expression. (B) CLR transformed read counts for HIF1A in ISAIAH patients, at d0, d28. Points represent patients; lines connect matched measurements from the same patient, where available. (C) Normalised enrichment score of cilia-related gene sets. (D) Matrix of genes significantly dysregulated between d0 and d28 and associated with >=2 gene sets identified in B. **** FDR<0.0001.

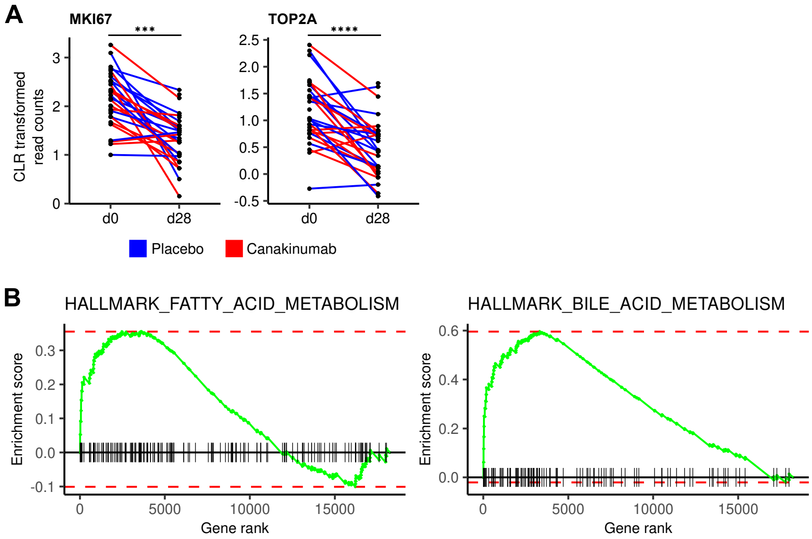

R1 Gut Figure S5

Supplementary Fig. 5: AH resolution reverses ALD-related proliferation and metabolism dysregulation.

(A) CLR transformed read counts for proliferation markers in ISAIAH patients (d0, d28). Points represent patients, with lines connecting matched measurements from the same patient, where available. (B) GSEA for the MSigDB Hallmark Fatty acid metabolism and Bile acid metabolism gene sets, comparing d28 and d0 gene expression. *** FDR<0.001; **** FDR<0.0001.

Rodrigo-Torres D, *et al. Gut* 2025;0:1–14. doi: 10.1136/gutjnl-2024-334094

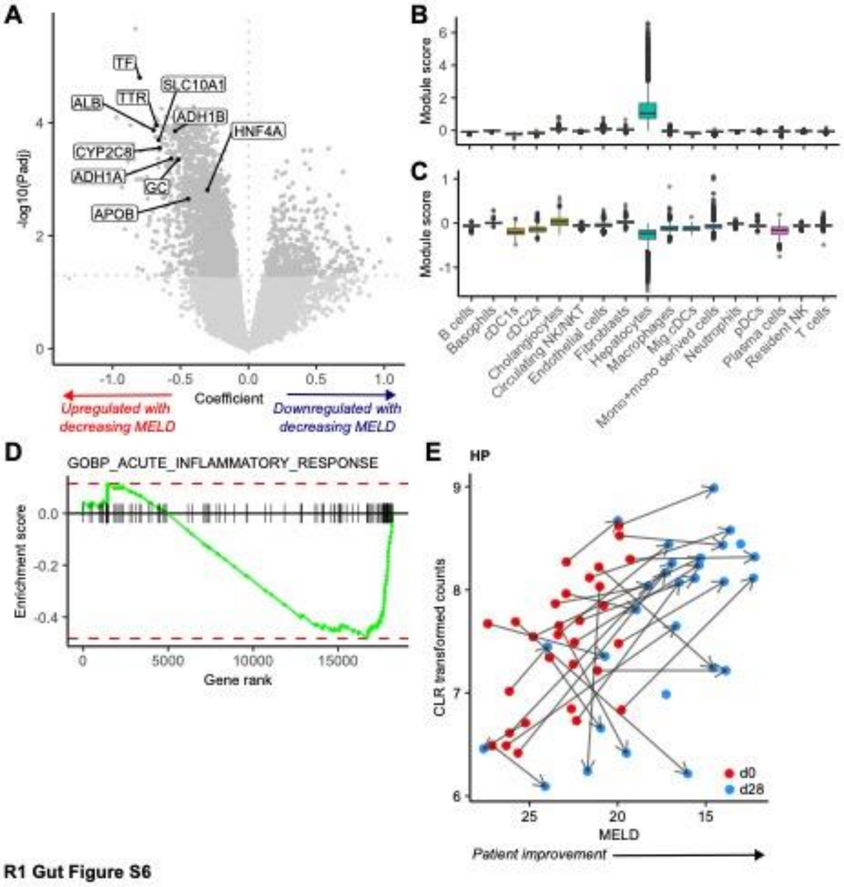

R1 Gut Figure S6

Supplementary Fig. 6: Hepatic and inflammatory dysregulation is reversed following AH resolution.

(A) Volcano plot of gene coefficient and significance with respect to MELD. Points represent genes; hepatocyte markers are highlighted. Module scores for genes significantly up- (B) and downregulated (C) with decreasing MELD. (D) GSEA for the Gene Ontology Acute inflammatory response gene set, using changes in gene expression with respect to MELD. (E) CLR transformed read counts for HP vs MELD, in ISAIAH patients, at d0 and d28. Points represent patients; arrows connect matched measurements from the same patient where available.

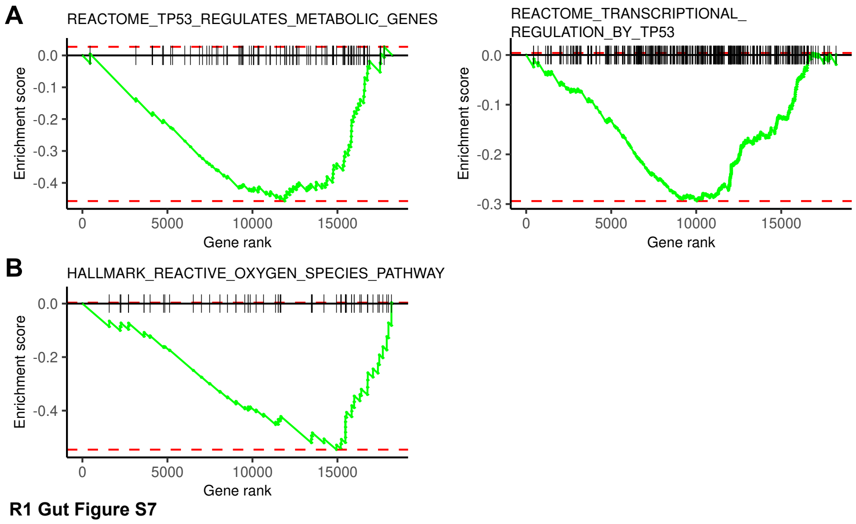

R1 Gut Figure S7

Supplementary Fig. 7: Senescence-related processes are disrupted by COX subunit dysregulation in AH.

(A) GSEA for the Reactome TP53 regulates metabolic genes and transcriptional regulation by TP53 gene sets, using changes in gene expression with respect to MELD. (B) GSEA for the Hallmark reactive oxygen species pathway gene set, using changes in gene expression with respect to MELD.

Rodrigo-Torres D, *et al. Gut* 2025;0:1–14. doi: 10.1136/gutjnl-2024-334094

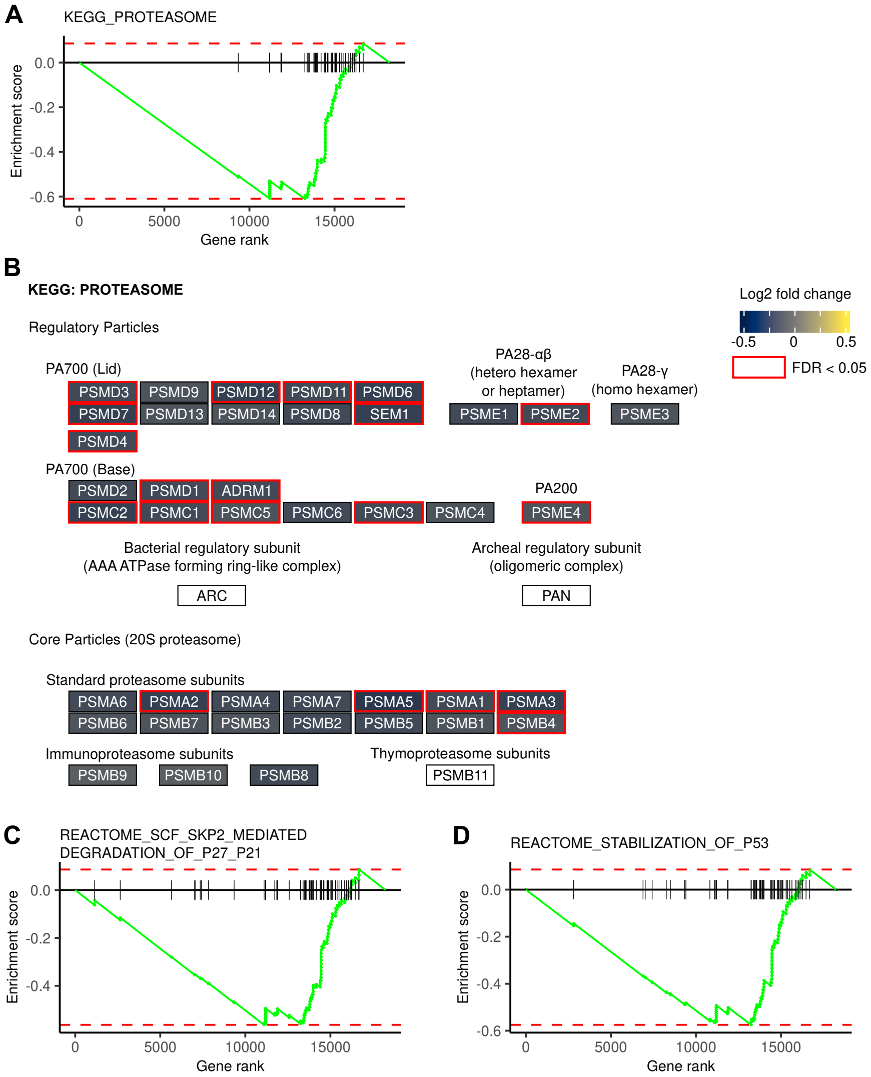

R1 Gut Figure S8

Supplementary Fig. 8: Senescence-related processes are disrupted by proteasomal dysregulation in AH.

(A) GSEA for the KEGG Proteasome gene set, using changes in gene expression with respect to MELD. (B) KEGG Proteasome pathway diagram. Significantly differentially expressed pathway members are coloured by coefficient change in d28, vs d0. GSEA for the Reactome (C) SCF(Skp2)-mediated degradation of p27/p21 and (D) Stabilization of P53 gene sets, using changes in gene expression with respect to MELD.

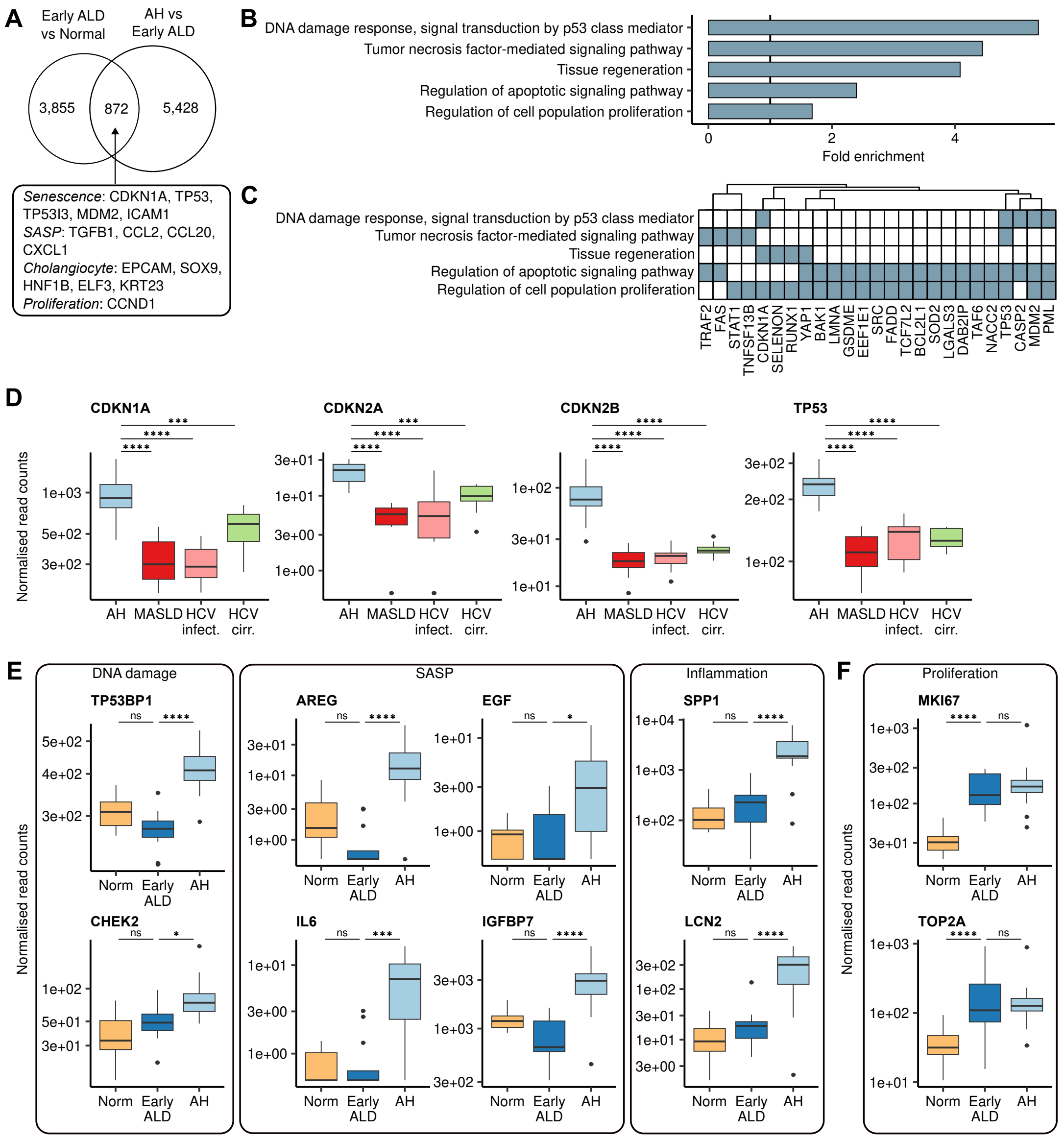

All data dbGAP: phs001807.v1.p1

R1 Gut Figure S9

Supplementary Fig. 9: A senescence signature is enriched in genes progressively upregulated with worsening ALD.

(A) Venn diagram of genes significantly upregulated in early ALD vs normal and in AH vs early ALD. (B) Fold enrichment of relevant Gene Ontology biological processes from overenrichment analysis of the n=872 intersection genes. (C) Matrix of intersection genes associated with >=2 pathways identified in B. (D) Boxplots of normalised read counts for senescence markers in AH (n=18), MASLD (n=9), chronic hepatitis C (HCV infect.; n=9) and compensated HCV cirrhosis (HCV cirr.; n=9). (E) Boxplots of normalised read counts for DNA damage, SASP, inflammation and (F) proliferation markers, in normal liver (*n*=10), early ALD (*n*=12) and AH (*n*=18). * FDR<0.05; *** FDR<0.001; **** FDR<0.0001.

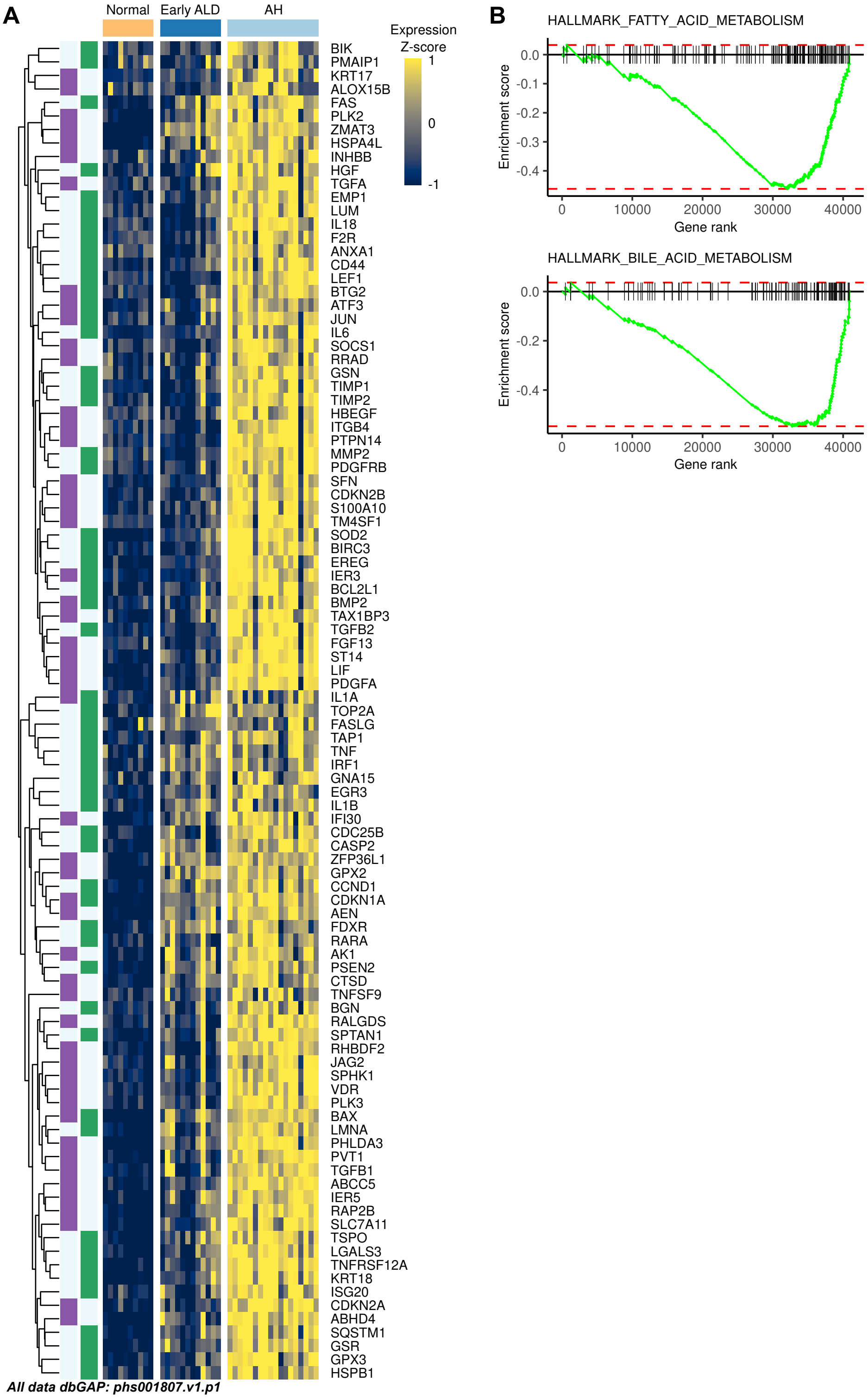

R1 Gut Figure S10

Supplementary Fig. 10: GSEA of AH vs normal liver reveals senescence and metabolic dysregulation.

(A) Heatmap of genes associated with the MSigDB Hallmark P53 pathway and Apoptosis gene sets in normal liver, early ALD and AH; all genes have significantly higher expression in AH vs normal liver. (B) GSEA for the MSigDB Hallmark Fatty acid metabolism and Bile acid metabolism gene sets, comparing AH to normal liver.

Rodrigo-Torres D, *et al. Gut* 2025;0:1–14. doi: 10.1136/gutjnl-2024-334094

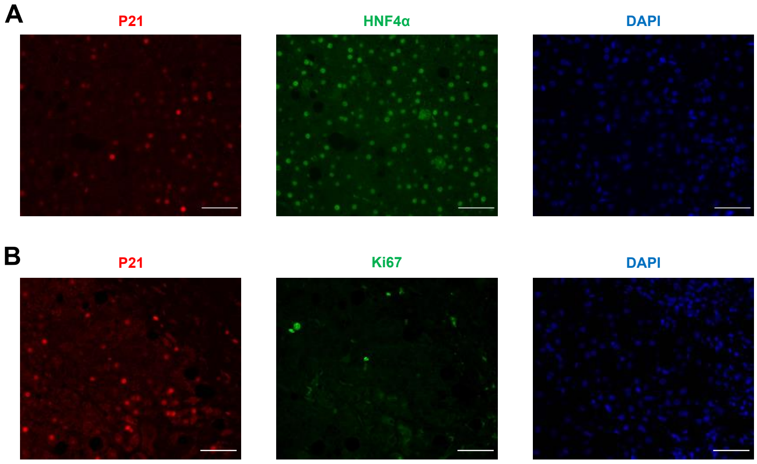

R1 Gut Figure S11

Supplementary Fig. 11: Single channels for Figure 4C and 4D in AH patients.

Dual immunofluorescence stainings of (A) p21 (red), HNF4α (green) and DAPI (blue); (B) p21 (red), Ki67 (green) and DAPI (blue). Scale bars: 100 μM.

Rodrigo-Torres D, *et al. Gut* 2025;0:1–14. doi: 10.1136/gutjnl-2024-334094

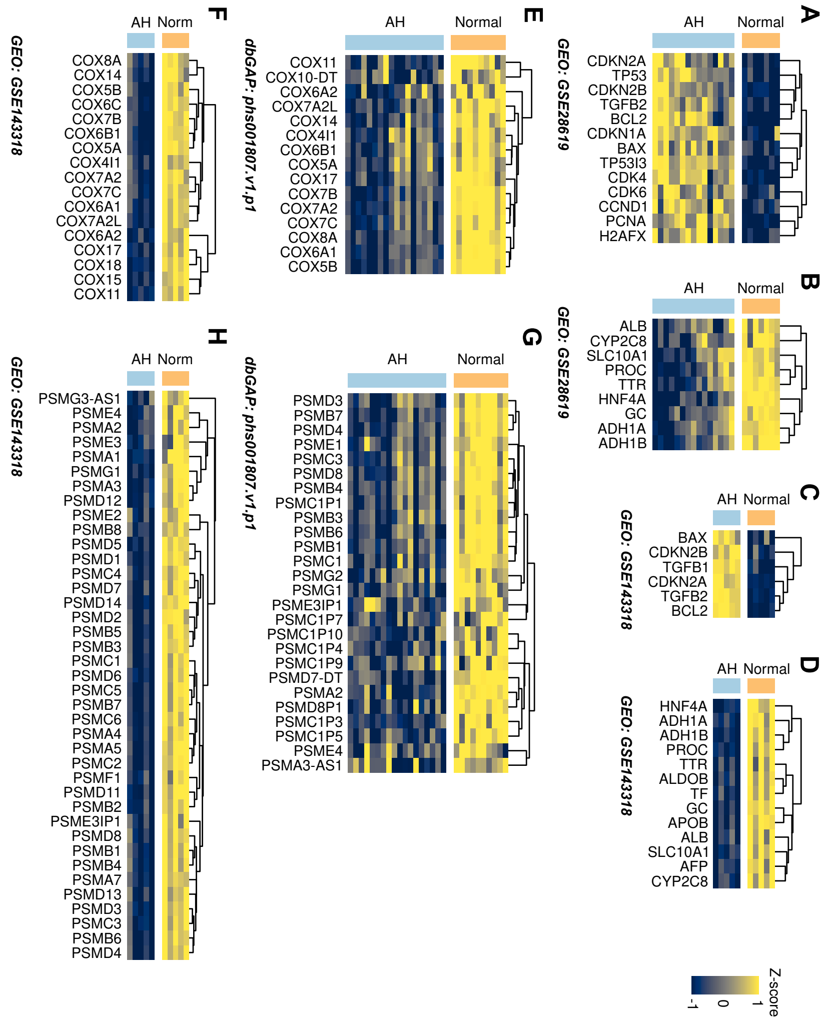

R1 Gut Figure S12

Supplementary Fig. 12: Senescence and related protein complex subunits are dysregulated in AH.

Heatmaps of (A) senescence markers and (B) hepatocyte markers significantly DE between normal liver (n=7) and AH (n=15); (C) senescence markers and (D) hepatocyte markers significantly DE between normal liver (n=5) and AH (n=5); (E) cytochrome c oxidase (COX) subunit genes significantly DE between normal liver (n=10) and AH (n=18); (F) COX subunit genes significantly DE between normal liver (n=5) and AH (n=5); (G) proteasome subunit genes significantly DE between normal liver (n=10) and AH (n=18); (H) proteasome subunit genes significantly DE between normal liver (n=5) and AH (n=5).

Rodrigo-Torres D, *et al. Gut* 2025;0:1–14. doi: 10.1136/gutjnl-2024-334094

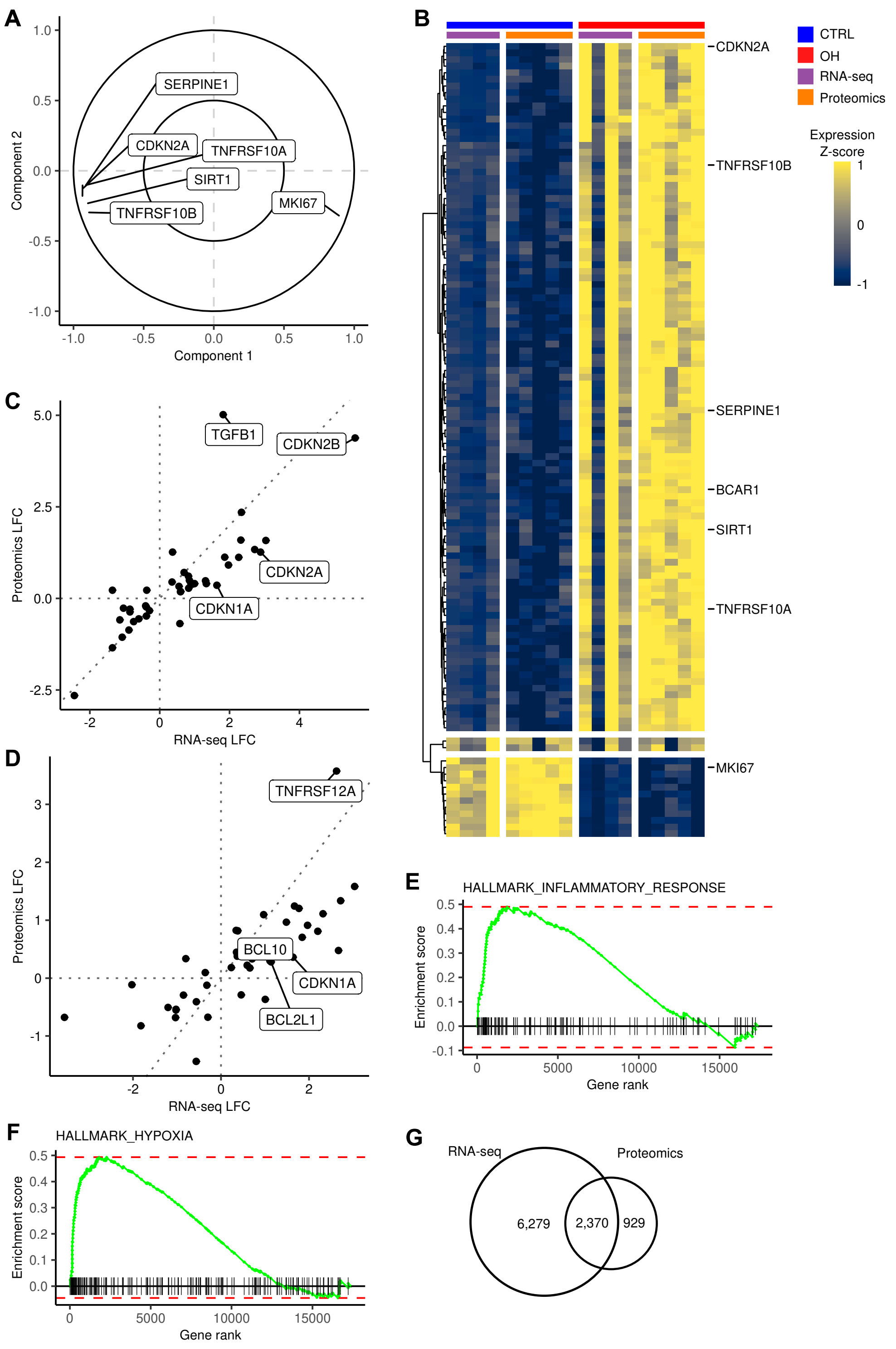

R1 Gut Figure S13

Supplementary Fig. 13: Ethanol induces a senescent response in the hepatocyte VL-17A cell line in multiomic analysis.

(A) Correlation plot of genomic features with correlation |>=0.9| to the first two components of the dimensionally reduced VL-17A multiomic dataset. (B) Heatmap of expression for features from A. Fold change in RNA-seq and proteomic analysis for (C) P53 pathway; and (D) apoptosis gene set members significantly DE in both analyses. (E) GSEA for the Hallmark inflammatory response; and (F) hypoxia gene sets, using changes in RNA-seq gene expression between untreated and EtOH-treated VL-17A cells. (G) Venn diagram of genes significantly DE in RNA-seq of VL-17A cells vs genes significantly DE in proteomic analysis.

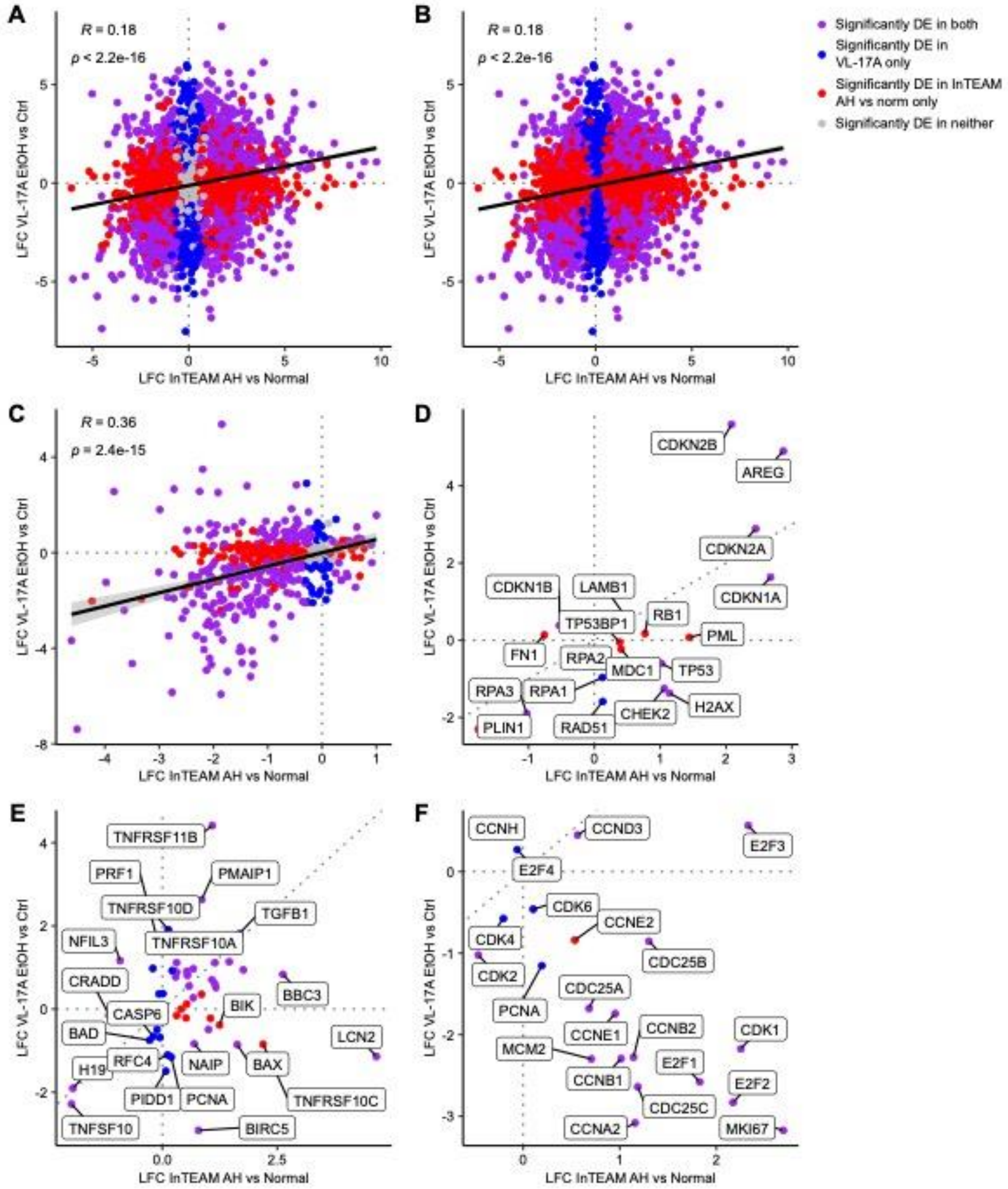

R1 Gut Figure S14

Rodrigo-Torres D, *et al. Gut* 2025;0:1–14. doi: 10.1136/gutjnl-2024-334094

Supplementary Fig. 14: Fold change direction between whole-liver and hepatocyte (VL-17A) cells is largely consistent.

Fold change in InTEAM AH vs normal RNA-seq data, compared to VL-17A EtOH-treated vs control cells, for (A) all matching genes; (B) genes significantly DE in at least one comparison; (C) high confidence hepatocyte-related genes; and panels of senescence- (D), apoptosis- (E) and proliferation-related (F) genes.

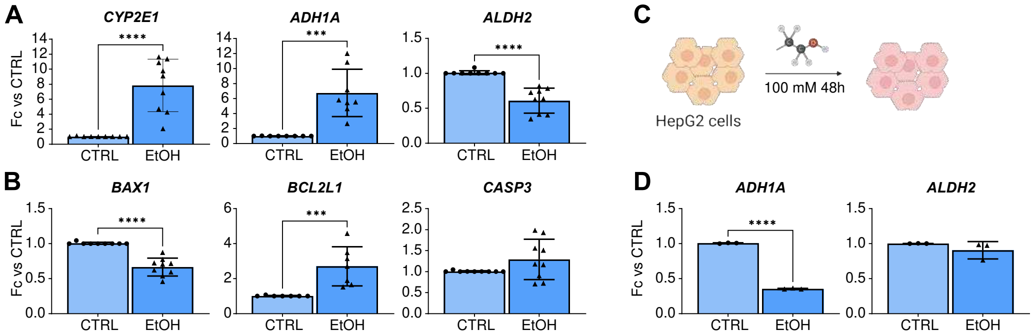

R1 Gut Figure S15

Supplementary Fig. 15: Ethanol metabolism is required for hepatocyte senescence induction in a hepatocyte (VL-17A) cell line.

Gene expression changes in (A) ethanol-metabolizing, and (B) apoptosis-related markers in VL-17A cells treated with 100mM ethanol (EtOH; n=9) for 48h versus untreated cells (CTRL; n=9). (C) Experimental design for in vitro treatment of HepG2 cells. (D) Differences in expression in ethanol-metabolization markers between untreated (CTRL; n=3) and ethanol-treated (EtOH; n=3) HepG2 cells. *** $p<0.001$; **** $p<0.0001$. (Mean ± SD). Fc: Fold change.

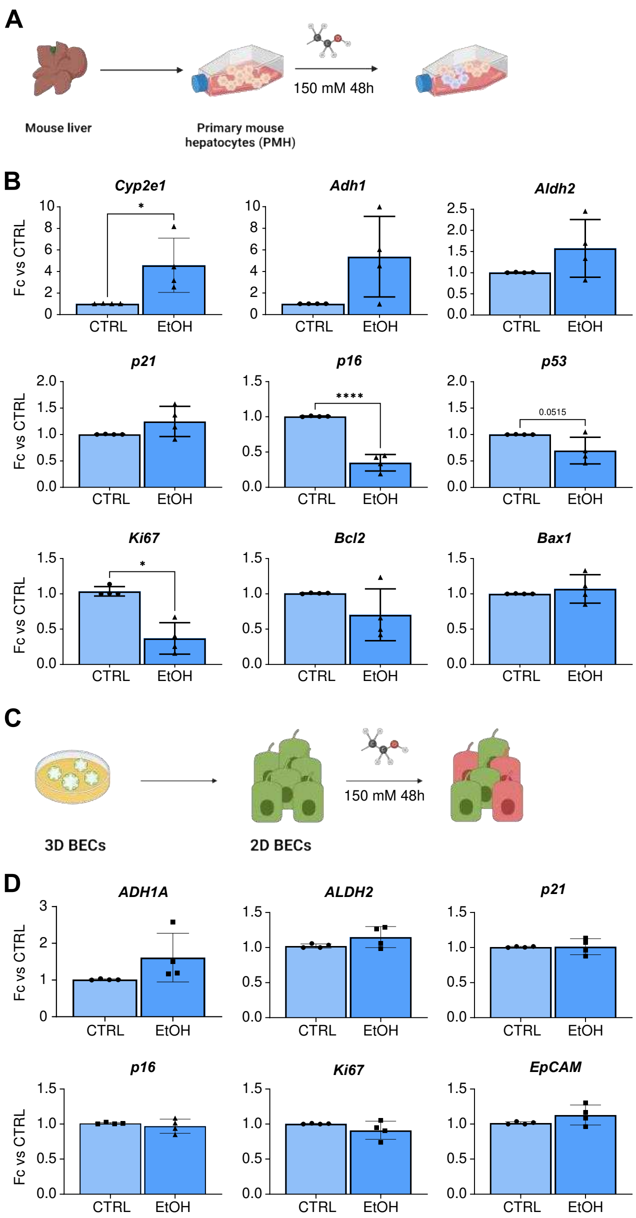

R1 Gut Figure S16

Supplementary Fig. 16: Effect of ethanol in other systems.

(A) Experimental design for *in vitro* ethanol treatment of primary mouse hepatocytes (PMH). (B) Gene expression differences between untreated (CTRL; n=4) and ethanol-treated (EtOH; n=4) PMH in ethanol-metabolising enzymes (*Cyp2e1*, *Adh1*, *Aldh2*), senescence (*p21*, *p16*, *p53*), proliferation (*Ki67*) and apoptosis (*Bax*, *Bcl2l1*) associated markers. (C) Schematic diagram of biliary epithelial cells (BECs) in vitro treatment with ethanol. (D) Expression differences between untreated (CTRL; n=4) and ethanol-treated BECs in genes associated with ethanol metabolism, senescence, proliferation and BECs identity. * $p< 0.05$, **** $p< 0.0001$. (Mean ± SD). Fc: Fold change.

Supplementary Tables

Supplementary Table 1. List of antibodies for immunostaining

Antibody	Company	Catalog Number	Species	Antigen Retrieval	Dilution
**Primary antibodies**					
γH2A.X	Abcam	ab81299	Rb mAb	T 15 min HP	1/2000
HNF4α	Bio-Techne	PP-H1415-0C	Ms mAb	T 15 min HP	1/200
Ki67	Abcam	Ab16667	Rb mAb	C 15 min HP	1/100
Ki67	Leica Biosystems	ACK02	Ms mAb	T 15 min HP	1/200
p21	DAKO	M7202	Ms mAb	T 10 min HP	1/20-1/40
p21	Cell Signaling	2947	Rb mAb	T 15 min HP	1/80
p53	Cell Signaling	48818	Ms mAb	C 15 min HP	1/50
**Secondary antibodies**					
Biotinylated Anti-Mouse IgG (H+L)	Vector Laboratories	BA-9200	Goat		1/500
Biotinylated Anti-Rabbit IgG (H+L)	Vector Laboratories	BA-1000	Goat		1/500
Biotinylated Ant-Rabbit IgG (H+L)	Vector Laboratories	BA-1100	Horse		1/500
Anti-Mouse IgG (H+L)- 488	Invitrogen	A21202	Donkey		1/200
Anti-Rabbit IgG (H+L)- 555	Invitrogen	A31572	Donkey		1/200
**Isotype controls**					
Mouse IgG	Vector Laboratories	I-2000	Mouse		Same concentration as primary antibody
Rabbit IgG	Vector Laboratories	I-1000	Rabbit		Same concentration as primary antibody

Ms, Mouse; Rb, Rabbit. mAb: monoclonal antibody.

Antigen retrieval: C, Citrate 1x; T, Tris-EDTA 1x (HP High power).

Rodrigo-Torres D, *et al. Gut* 2025;0:1–14. doi: 10.1136/gutjnl-2024-334094

Supplementary Table 2. Primers for RT-qPCR (Qiagen QuantiTect Primer Assay).

Name	Species	Ref#
ADH1A	human	QT00055188
Adh1	mouse	QT00093520
ALDH2	human	QT00058093
Aldh2	mouse	QT00158368
BAX1	human	QT00031192
Bax1	mouse	QT00102536
BCL2L1	human	QT00236712
Bcl2l1	mouse	QT00149254
CDKN1A	human	QT00062090
Cdkn1a	mouse	QT00137053
CDKN2A	human	QT00089964
Cdkn2a	mouse	QT00252595
Cxcl1	mouse	QT00115647
CYP2E1	human	QT00004382
Cyp2e1	mouse	QT00112539
EpCAM	human	QT00000371
Lcn2	mouse	QT00113407
MKi67	human	QT00014203
Mki67	mouse	QT00247667
Trp53	mouse	QT00101906

Supplementary Table 3. Differential expression of senescence markers in different datasets.

	ISAIAH d28 vs d0		INTEAM AH vs Normal		VL-17A ETOH vs CTRL	
	log2FoldChange	padj	log2FoldChange	padj	log2FoldChange	padj
**CDKN2B**	-0.81	1.80E-05	2.13	3.87E-21	5.59	3.39E-128
**AREG**	-1.12	1.45E-04	2.92	2.84E-05	4.90	1.64E-25
**CDKN2A**	-0.62	2.11E-02	2.50	5.94E-20	2.89	1.18E-60
**CDKN1A**	-0.51	1.91E-02	2.72	8.27E-44	1.64	9.98E-16
**PML**	-0.13	1.04E-01	1.49	9.95E-16	0.08	7.00E-01
**RB1**	-0.21	1.30E-02	0.82	1.32E-11	0.17	2.82E-01
**LAMB1**	-0.15	2.70E-02	0.59	5.21E-04	0.47	1.95E-05
**TP53BP1**	-0.21	3.11E-03	0.44	1.95E-07	-0.05	7.54E-01
**CDKN1B**	-0.07	5.97E-01	-0.48	1.56E-05	0.38	5.08E-03
**RPA3**	-0.08	6.51E-01	-0.98	8.40E-14	-1.90	4.93E-25
**PLIN1**	0.08	7.93E-01	-1.70	3.63E-07	-2.30	1.45E-01
**RAD51**	-0.46	4.45E-02	0.18	6.12E-01	-1.58	3.20E-08
**RPA1**	-0.03	6.32E-01	0.17	2.65E-02	-0.96	8.93E-14
**RPA2**	-0.07	3.82E-01	-0.37	1.23E-02	-0.30	1.08E-01
**MDC1**	0.04	8.04E-01	0.46	3.39E-03	-0.23	1.12E-01
**CHEK2**	-0.28	9.43E-03	1.11	5.89E-05	-1.25	1.02E-11
**H2AX**	-0.17	2.36E-01	1.19	1.32E-10	-1.37	7.14E-20
**TP53**	-0.13	1.17E-01	1.07	6.98E-25	-0.60	5.95E-05

Supplementary Table 4. Differential expression of apoptosis markers in different datasets.

	ISAIAH d28 vs d0		INTEAM AH vs Normal		VL-17A ETOH vs CTRL	
	log2FoldChange	padj	log2FoldChange	padj	log2FoldChange	padj
TNFRSF10C	-0.51	2.17E-04	2.25	1.60E-10	-0.85	1.57E-01
LCN2	-1.75	2.12E-07	4.71	5.46E-23	-1.14	4.25E-02
BAX	-0.32	1.13E-02	1.68	1.13E-28	-0.85	1.40E-13
BBC3	-0.20	8.37E-02	2.67	3.55E-31	0.83	2.84E-08
TGFB1	-0.02	8.40E-01	1.72	8.67E-14	1.82	8.99E-36
PMAIP1	-0.52	5.85E-02	0.89	9.84E-03	2.63	2.11E-25
TNFRSF11B	-0.24	1.51E-01	1.13	1.09E-08	4.41	6.38E-04
PRF1	0.18	1.76E-01	-0.06	8.99E-01	1.69	1.75E-07
TNFRSF10D	-0.24	4.58E-02	0.18	4.00E-01	1.91	8.35E-35
TNFRSF10A	-0.25	3.60E-02	1.28	1.56E-11	1.63	5.86E-27
BIK	-0.49	1.10E-01	1.32	2.01E-03	-0.39	2.71E-01
NAIP	-0.21	4.74E-03	0.75	9.36E-07	-0.83	1.18E-03
PIDD1	-0.06	7.37E-01	0.14	6.67E-01	-1.49	7.65E-10
RFC4	-0.09	5.29E-01	0.17	3.20E-01	-1.10	5.48E-11
TNFSF10	0.22	1.21E-01	-1.93	6.75E-16	-2.28	5.19E-15
H19	0.38	8.39E-02	-1.89	3.53E-05	-1.91	1.08E-08
BAD	-0.05	7.04E-01	-0.23	1.24E-01	-0.75	1.72E-05
CASP6	-0.14	1.64E-01	-0.15	2.96E-01	-0.66	9.71E-05
CRADD	0.10	2.61E-01	-1.10	1.85E-46	-0.15	5.29E-01
NFIL3	-0.39	2.62E-03	-0.87	5.81E-04	1.16	3.41E-17

Rodrigo-Torres D, *et al. Gut* 2025;0:1–14. doi: 10.1136/gutjnl-2024-334094

Supplementary Table 5. Differential expression of proliferation markers in different datasets.

	ISAIAH d28 vs d0		INTEAM AH vs Normal		VL-17A ETOH vs CTRL	
	log2FoldChange	padj	log2FoldChange	padj	log2FoldChange	padj
CCNH	0.01	9.45E-01	-0.15	1.88E-01	0.50	3.54E-04
CCND3	-0.11	2.73E-01	0.62	5.90E-06	0.45	3.11E-02
E2F3	-0.39	3.46E-04	2.38	2.01E-70	0.57	6.86E-03
E2F4	-0.12	8.52E-02	-0.01	9.49E-01	0.27	4.65E-02
CDK6	-0.23	1.89E-02	0.15	3.74E-01	-0.46	8.71E-06
CDK4	-0.07	6.02E-01	-0.15	2.27E-01	-0.58	1.56E-07
CCNE2	-0.55	3.81E-02	0.58	1.77E-03	-0.84	1.00E-01
CDK2	-0.05	5.22E-01	-0.41	6.92E-05	-1.02	3.05E-10
CDC25A	-0.37	7.78E-02	0.72	4.48E-02	-1.68	9.56E-13
CDC25B	-0.18	3.67E-02	1.36	2.61E-13	-0.85	9.97E-08
PCNA	-0.17	2.65E-01	0.24	2.37E-01	-1.15	8.56E-13
MCM2	0.05	7.62E-01	0.76	7.27E-03	-2.30	3.80E-47
CCNE1	-0.47	4.51E-02	1.02	9.59E-04	-1.74	6.11E-31
CCNB2	-0.48	3.51E-03	1.20	1.75E-04	-2.28	1.62E-14
CDK1	-0.58	1.78E-02	2.31	4.83E-10	-2.17	2.08E-06
CCNB1	-0.48	5.74E-03	1.07	1.36E-03	-2.29	4.40E-24
E2F1	-0.18	2.88E-01	1.88	5.43E-06	-2.58	2.93E-54
E2F2	-0.42	9.17E-02	2.23	2.05E-06	-2.83	1.95E-31
CCNA2	-0.48	3.99E-03	1.21	1.72E-04	-3.08	2.25E-06
MKI67	-0.81	1.40E-05	2.74	2.41E-18	-3.17	4.88E-11

Supplementary Table 6. Differential expression of hepatocyte markers in different datasets.

	ISAIAH d28 vs d0		INTEAM AH vs Normal		VL-17A ETOH vs CTRL	
	**log2FoldChange**	**padj**	**log2FoldChange**	**padj**	**log2FoldChange**	**padj**
**ALB**	0.81	7.75E-04	-2.21	1.97E-13	-1.58	9.26E-39
**ADH1A**	0.70	5.00E-04	-3.15	1.40E-20	-	-
**CYP2E1**	0.06	8.70E-01	-3.78	4.29E-26	2.54	1.59E-40
**TTR**	1.08	1.80E-05	-3.60	8.42E-34	-2.43	1.68E-46
**TF**	0.85	3.71E-04	-1.92	6.37E-09	-2.58	7.45E-53
**ADH1B**	0.57	2.73E-03	-2.87	7.06E-21	-	-
**SLC10A1**	0.56	1.85E-02	-2.08	7.98E-19	-	-
**HNF4A**	0.24	4.19E-02	-0.36	2.20E-02	-0.58	8.76E-10
**ALDOB**	0.68	7.19E-04	-2.46	1.25E-27	-1.48	4.61E-02

* ADH1A, ADH1B, SLC10A1 filtered due to low expression

Supplementary References

1. Gaitantzi H, Meyer C, Rakoczy P, et al. Ethanol sensitizes hepatocytes for TGF-β-triggered apoptosis. Cell Death Dis. 2018;9(2):51. https://doi.org/10.1038%2Fs41419-017-0071-y

2. Huch M, Gehart H, van Boxtel R, et al. Long-term culture of genome-stable bipotent stem cells from adult human liver. Cell. 2015;160(1-2):299-312. https://doi.org/10.1016/j.cell.2014.11.050

3. Gaitantzi H, Meyer C, Rakoczy P, et al. Ethanol sensitizes hepatocytes for TGF-β-triggered apoptosis. Cell Death Dis. 2018;9(2):51. https://doi.org/10.1038/s41419-017-0071-y

4. Argemi J, Latasa MU, Atkinson SR, et al. Defective HNF4alpha-dependent gene expression as a driver of hepatocellular failure in alcoholic hepatitis. Nature Communications. 2019; 10:3126. https://doi.org/10.1038/s41467-019-11004-3

5. Hyun J, Sun Z, Ahmadi AR, et al. Epithelial splicing regulatory protein 2-mediated alternative splicing reprograms hepatocytes in severe alcoholic hepatitis. Journal of Clinical Investigation. 2020; 130(4):2129–2145. https://doi.org/10.1172/JCI132691

6. Martin M. Cutadapt removes adapter sequences from high-throughput sequencing reads. EMBnet.journal. 2011; 17(1):10-12. https://doi.org/10.14806/ej.17.1.200

7. Dobin A, Davis CA, Schlesinger F, et al. STAR: ultrafast universal RNA-seq aligner. Bioinformatics. 2013; 29(1):15-21 https://doi.org/10.1093/bioinformatics/bts635

8. Li, B., Dewey, C.N. RSEM: accurate transcript quantification from RNA-Seq data with or without a reference genome. BMC Bioinformatics 12, 323 (2011). https://doi.org/10.1186/1471-2105-12-323

9. Thévenot EA, Roux A, Xu Y, Ezan E, Junot C. Analysis of the Human Adult Urinary Metabolome Variations with Age, Body Mass Index, and Gender by Implementing a Comprehensive Workflow for Univariate and OPLS Statistical Analyses. Journal of Proteome Research 2015; 14(8):3322-3335 https://doi.org/10.1021/acs.jproteome.5b00354

10. Robinson MD, McCarthy DJ, Smyth GK. edgeR: a Bioconductor package for differential expression analysis of digital gene expression data. Bioinformatics 2010; 26(1):139-140. https://doi.org/10.1093/bioinformatics/btp616

11. Love MI, Huber W, Anders S. Moderated estimation of fold change and dispersion for RNA-seq data with DESeq2. Genome Biology. 2014; 15(550). https://doi.org/10.1186/s13059-014-0550-8

12. Martinez-Gili L, Pechlivanis A, McDonald JAK, et al. Bacterial and metabolic phenotypes associated with inadequate response to ursodeoxycholic acid treatment in primary biliary cholangitis. Gut Microbes 2023; 15(1): 2208501. https://doi.org/10.1080%2F19490976.2023.2208501

13. Twisk J, Bosman L, Hoekstra T, et al. Different ways to estimate treatment effects in randomised controlled trials. Contemporary Clinical Trials Communications 2018; 10:80-85. https://doi.org/10.1016/j.conctc.2018.03.008

14. Gu Z, Eils R, Schlesner M. Complex heatmaps reveal patterns and correlations in multidimensional genomic data. Bioinformatics, 2016; 32(18):2847-2849. https://doi.org/10.1093/bioinformatics/btw313

15. Perez-Riverol Y, Bai J, Bandla C, et al. (2022). The PRIDE database resources in 2022: A Hub for mass spectrometry-based proteomics evidences. Nucleic Acids Res 2022. 50(D1):D543-D552. https://doi.org/10.1093/nar/gkab1038

16. **Krotkevich G, Sukhov V**, Budin N, et al. Fast gene set enrichment analysis. biorXiv 060012. https://doi.org/10.1101/060012

17. Liberzon A, Birger C, Thorvaldsdóttir H, et al. The Molecular Signatures Database Hallmark Gene Set Collection. Cell Systems 2015 1(6):P417-425 https://doi.org/10.1016/j.cels.2015.12.004

18. Luo W, Brouwer C. Pathview: an R/Bioconductor package for pathway-based data integration and visualization. Bioinformatics. 2013; 29(14):1830-1831. https://doi.org/10.1093/bioinformatics/btt285

19. Rohart F, Gautier B, Singh A, Lê Cao KA. mixOmics: An R package for ‘omics feature selection and multiple data integration. PLOS Computational Biology 2017; 13(11): e1005752. https://doi.org/10.1371/journal.pcbi.1005752

20. Odom GJ, Colaprico A, Silva TC, et al. PathwayMultiomics: An R Package for Efficient Integrative Analysis of Multi-Omics Datasets With Matched or Un-matched Samples. Frontiers in Genetics 2021; 12 https://doi.org/10.3389/fgene.2021.783713

21. Affò S, Dominguez M, Lozano JJ, et al. Transcriptome analysis identifies TNF superfamily receptors as potential therapeutic targets in alcoholic hepatitis. Gut. 2013; 62:452-460. https://doi.org/10.1136/gutjnl-2011-301146

22. Ritchie ME, Phipson B, Wu D, et al. Limma powers differential expression analyses for RNA-sequencing and microarray studies. Nucleic Acids Research 2015; 43(7):e47. https://doi.org/10.1093/nar/gkv007

23. Guilliams M, Bonnardel J, Haest B, et al. Spatial proteogenomics reveals distinct and evolutionarily conserved hepatic macrophage niches. Cell. 2022; 185(2): P379-396.e38. https://doi.org/10.1016/j.cell.2021.12.018

24. **Hao Y, Hao S,** Andersen-Nissen E, et al. Integrated analysis of multimodal single-cell data. Cell. 2021; 184, 3573-3587. https://doi.org/10.1016/j.cell.2021.04.048

25. Guan Y, Peiffer B, Feng D et al. IL-8+ neutrophils drive inexorable inflammation in severe alcohol-associated hepatitis. J Clin Invest. 2024; 134(9):e178616. http://doi.org/10.1172/JCI178616

26. Zeng D, Fang Y, Qiu W, et al. Enhancing immuno-oncology investigations through multidimensional decoding of tumor microenvironment with IOBR 2.0. Cell Rep Methods. 2024; 4(12):100910. https://doi.org/10.1016/j.crmeth.2024.100910

27. Newman AM, Liu CL, Green MR, et al. Robust enumeration of cell subsets from tissue expression profiles. Nat Methods. 2015; 12(5):453-457. https://doi.org/10.1038/nmeth.3337
